# Exploring Variability of *Trichodesmium* Photophysiology Using Multi-Excitation Wavelength Fast Repetition Rate Fluorometry

**DOI:** 10.3389/fmicb.2022.813573

**Published:** 2022-04-08

**Authors:** Yuanli Zhu, Yuanyuan Feng, Thomas J. Browning, Zuozhu Wen, David J. Hughes, Qiang Hao, Ruifeng Zhang, Qicheng Meng, Mark L. Wells, Zhibing Jiang, P. A. K. N. Dissanayake, W. N. C. Priyadarshani, Lu Shou, Jiangning Zeng, Fei Chai

**Affiliations:** ^1^State Key Laboratory of Satellite Ocean Environment Dynamics, Second Institute of Oceanography, Ministry of Natural Resources, Hangzhou, China; ^2^Key Laboratory of Marine Ecosystem Dynamics, Second Institute of Oceanography, Ministry of Natural Resources, Hangzhou, China; ^3^School of Oceanography, Shanghai Jiao Tong University, Shanghai, China; ^4^Marine Biogeochemistry Division, GEOMAR Helmholtz Centre for Ocean Research, Kiel, Germany; ^5^State Key Laboratory of Marine Environmental Science, Xiamen University, Xiamen, China; ^6^Climate Change Cluster, Faculty of Science, University of Technology Sydney, Ultimo, NSW, Australia; ^7^Darling Marine Center, University of Maine, Walpole, ME, United States; ^8^Department of Oceanography and Marine Geology, Faculty of Fisheries and Marine Sciences and Technology, University of Ruhuna, Matara, Sri Lanka; ^9^National Institute of Oceanography and Marine Sciences, National Aquatic Resources Research and Development Agency, Colombo, Sri Lanka

**Keywords:** *Trichodesmium*, fast repetition rate fluorometer, photophysiology, photobiology, photoacclimatiion, iron stress, nutrient limitation

## Abstract

Fast repetition rate fluorometry (FRRf) allows for rapid non-destructive assessment of phytoplankton photophysiology *in situ* yet has rarely been applied to *Trichodesmium.* This gap reflects long-standing concerns that *Trichodesmium* (and other cyanobacteria) contain pigments that are less effective at absorbing blue light which is often used as the sole excitation source in FRR fluorometers—potentially leading to underestimation of key fluorescence parameters. In this study, we use a multi-excitation FRR fluorometer (equipped with blue, green, and orange LEDs) to investigate photophysiological variability in *Trichodesmium* assemblages from two sites. Using a multi-LED measurement protocol (447+519+634 nm combined), we assessed maximum photochemical efficiency (*F*_*v*_/*F*_*m*_), functional absorption cross section of PSII (σ_*PSII*_), and electron transport rates (ETRs) for *Trichodesmium* assemblages in both the Northwest Pacific (NWP) and North Indian Ocean in the vicinity of Sri Lanka (NIO-SL). Evaluating fluorometer performance, we showed that use of a multi-LED measuring protocol yields a significant increase of *F*_*v*_/*F*_*m*_ for *Trichodesmium* compared to blue-only excitation. We found distinct photophysiological differences for *Trichodesmium* at both locations with higher average *F*_*v*_/*F*_*m*_ as well as lower σ_*PSII*_ and non-photochemical quenching (*NPQ*_*NSV*_) observed in the NWP compared to the NIO-SL (Kruskal–Wallis *t*-test *df* = 1, *p* < 0.05). Fluorescence light response curves (FLCs) further revealed differences in ETR response with a lower initial slope (α_*ETR*_) and higher maximum electron turnover rate ($ETR_{PSII}^{max}$) observed for *Trichodesmium* in the NWP compared to the NIO-SL, translating to a higher averaged light saturation *E*_*K*_ (= $ETR_{PSII}^{max}$/α_*ETR*_) for cells at this location. Spatial variations in physiological parameters were both observed between and within regions, likely linked to nutrient supply and physiological stress. Finally, we applied an algorithm to estimate primary productivity of *Trichodesmium* using FRRf-derived fluorescence parameters, yielding an estimated carbon-fixation rate ranging from 7.8 to 21.1 mgC mg Chl-a^–1^ h^–1^ across this dataset. Overall, our findings demonstrate that capacity of multi-excitation FRRf to advance the application of Chl-a fluorescence techniques in phytoplankton assemblages dominated by cyanobacteria and reveals novel insight into environmental regulation of photoacclimation in natural *Trichodesmium* populations.

## Introduction

Fast repetition rate fluorometry (FRRf) is sensitive enough for use in low-chlorophyll *a* oligotrophic waters and provides a non-destructive and minimally intrusive method for probing photosynthetic processes, including photosystem II (PSII) photochemistry and photosynthetic electron transport (Kolber et al., 1998; Schuback et al., 2021). This technique is now an established tool in global aquatic research efforts to understand environmental regulation of phytoplankton physiology and productivity (Gorbunov et al., 1999, 2001; Suggett et al., 2009a; Hughes et al., 2018a; Schuback et al., 2021). FRRf observations of phytoplankton have been used principally to examine the effects of physiological stress, such as nutrient limitation (Behrenfeld et al., 2006; Schuback et al., 2016; Hughes et al., 2018b). Recently, FRRf has also been used for the characterization of light absorption (Silsbe et al., 2015), for interpreting phytoplankton photophysiological processes in the context of phytoplankton community structure (Carvalho et al., 2020; Gorbunov et al., 2020; Hughes et al., 2020), and for describing primary productivity (e.g., Smyth et al., 2004; Fujiki et al., 2008; Cheah et al., 2011; Wei et al., 2020; Zhu et al., 2016, 2017, 2019; Hughes et al., 2018b; Schuback et al., 2021).

This has even extended to autonomous deployment across all major oceans *via* research vessels, buoy systems, and glider platforms (Falkowski et al., 2017; Carvalho et al., 2020; Ryan-Keogh and Thomalla, 2020).

As blue light is both high in energy and strongly absorbed by chlorophyll, until recently the majority of fluorometers were equipped with only blue-excitation LEDs (usually with wavelengths between 450 and 470 nm). Critically, measurements of PSII maximum photochemical efficiency (*F*_*v*_/*F*_*m*_) and functional absorption cross section of PSII (σ_*PSII*_)—which are related to photosynthetic performance (see Hughes et al., 2018a; Schuback et al., 2021)—are inherently scaled to the excitation LED of the instrument. However, it has long been known that blue-light equipped FRRf instruments are relatively insensitive to the presence of cyanobacteria—which absorb light in the blue region extremely poorly compared to other phytoplankton groups (Raateoja et al., 2004; Suggett et al., 2004). Cyanobacteria such as *Trichodesmium* exhibit a very low σ_*PSII*_ at 400–500 nm, instead absorbing strongly in the green or orange/red regions due to their use of phycobilisomes rich in phycocyanin or long-wavelength variants of phycoerythrin (Houliez et al., 2017). As such, FRRf instruments containing only a single blue-excitation LED often fail to adequately drive PSII reaction center closure in cyanobacterial samples (e.g., Hughes et al., 2020), generally resulting in underestimation of electron transport rates (ETRs) (Robinson et al., 2014). To this end, multi-excitation fluorometers have emerged in recent years, facilitating measurement of phytoplankton communities dominated by cyanobacteria, and to date have been successfully utilized in both marine and freshwater systems (Simis et al., 2012; Houliez et al., 2017; Wei et al., 2019; Kazama et al., 2021).

Our interest here is focused on identifying the physiological ecology of *Trichodesmium* in oligotrophic waters using multiwavelength FRRf measurements. The diazotrophic cyanobacterium *Trichodesmium* is a major contributor to new nitrogen (N) production in parts of the oligotrophic subtropical and tropical ocean, with large surface accumulations of *Trichodesmium* reported episodically (Capone et al., 2005). Such *Trichodesmium* accumulations often result in transient domination of overall phytoplankton primary productivity (Bowman and Lancaster, 1965; Capone et al., 1997; Karl et al., 1997). As such, *Trichodesmium* plays a critical role in the biogeochemical cycling of C and N in oligotrophic regions.

*Trichodesmium* exhibits a high PSI:PSII ratio, which minimizes damage to nitrogenase from photosynthetic O_2_ production at PSII, yet also makes *Trichodesmium* difficult to detect by chlorophyll fluorescence (Subramaniam et al., 1999). Furthermore, *Trichodesmium* possess phycobiliproteins with absorption peaks centered at 495, 545, and 565 nm (Fujita and Shimura, 1974). As a consequence of the combination of the phycobiliproteins and a relatively low abundance of PSII, lower values of both *F*_*v*_/*F*_*m*_ and σ_*PSII*_ have been documented for *Trichodesmium* when using fluorometers with solely blue-excitation sources (Campbell et al., 1998; Raateoja et al., 2004; Cai et al., 2015). Consequently, consideration of the excitation wavelength is crucial when interpreting *in situ* photophysiological data (Suggett et al., 2009b).

In this study, we present the results of the first study using an FRR fluorometer equipped with multi-excitation wavelength LEDs to assess the photobiology of natural *Trichodesmium* populations from two water bodies, the Northwest Pacific (NWP) and North Indian Ocean in the vicinity of Sri Lanka (NIO-SL). The primary objective was to accurately characterize the two photosynthetic parameters describing the physiological state of PSII (*F*_*v*_/*F*_*m*_ and σ_*PSII*_) for *Trichodesmium*. First, we evaluated the performance of FRRf with multi-excitation wavelengths and documented the improvement of retrieving photophysical parameters when using the combined measurement protocol rather than using blue light only. We then compared photophysical parameters of *Trichodesmium* between and within study regions, evaluating the importance of potential nutrient stress (in this case mainly iron) on driving variations of *F*_*v*_/*F*_*m*_ and σ_*PSII*_. Finally, we estimated the carbon uptake rate of *Trichodesmium* from FRR data, highlighting the possibility of direct measurement of primary production of *Trichodesmium* in the field from *in-situ* fluorometric data.

## Materials and Methods

### Water Sample Collection and Physical and Biochemical Properties

**FIGURE 1:**
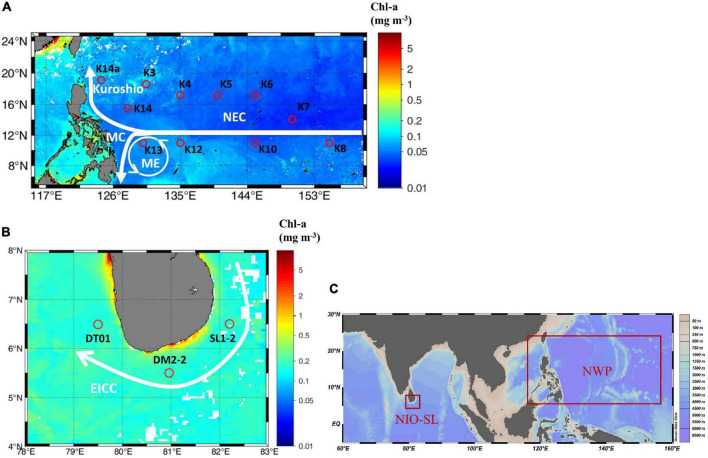
**(A)** Sampling stations during the GEOTRACES cruise GP09 performed onboard the R/V *Tan Kah Kee* during the spring (April 25–June 13, 2019) in the Northwest Pacific (NWP), and **(B)** during an international joint cruise of the R/V *Xiangyanghong-6* during January 2020 in the North Indian Ocean in the vicinity of Sri Lanka (NIO-SL). Main currents are also shown, which include Kuroshio, North Equatorial Current (NEC), Mindanao Current (MC), and Mindanao Eddy (ME) in the NWP (Liu et al., 2017) as well as East Indian Coast Current (EICC) in the NIO-SL (Vos et al., 2014). **(C)** Global locations of two study regions. The figure’s background is Moderate Resolution Imaging Spectroradiometer (MODIS) monthly Level 3 Chl-a data which were downloaded from NASA’s ocean color website (http://oceandata.sci.gsfc.nasa.gov).

Sampling was conducted aboard the GEOTRACES cruise GP09 of the R/V *Tan Kah Kee* during the spring (April 25–June 13, 2019) in the NWP, and during an international joint cruise of the R/V *Xiangyanghong-6* in January 2020 in the NIO-SL (Figure 1). All shipboard measurements were performed by the same operator, with identical protocols adopted for handling and processing of samples in both NWP and NIO-SL cruises. Routine water samples of biological cast were collected using a rosette equipped with 12 Niskin bottles (10-l capacity; General Oceanics Inc, Miami, FL, United States) and a conductivity-temperature-depth profiler (911+, Sea-Bird Electronics, Bellevue, WA, United States). Sampling was performed in the morning between 5:00 and 8:00 a.m. local time (Table 1). The upper mixed layer depth (MLD) was defined as a density change from the ocean surface of 0.125 sigma units (Huang and Russell, 1994). Incident surface photosynthetically active radiation (PAR, 400–700 nm, measured in μmol quanta m^–2^ s^–1^) was measured throughout the cruise period with a quantum scalar irradiance sensor (QSL-2100, Biospherical Instruments Inc, San Diego, CA, United States).

**TABLE 1:** Surface values of temperature (Temp, °C), salinity (Sal), mixed layer depth (MLD, m), Chl-a concentration (Chl-a, μg L^–1^), PAR (mol photon m^–2^ d^–1^), nitrite and nitrate (NO_2−_ + NO_3–_, nmol L^–1^) and phosphate (PO_43–_, nmol L^–1^) in the NWP and NIO-SL.

Region	Stn.	Lon.	Lat.	Sampling date	Local time	Temp	Sal.	MLD	Chl-a	PAR	NO_2_ + NO_3–_	PO_43–_	N/P
NWP	K3	130.42	18.6	2019-05-03	05:11	28.8	34.6	16	0.04	60.7	BLD	27.1	N/A
	K4	135	17.2	2019-05-20	06:08	29.8	34.6	23	0.05	51.6	BLD	26.5	N/A
	K5	140	17.2	2019-05-21	06:19	29.3	34.2	16	0.07	56.4	13.2	32.7	0.40
	K6	145	17.2	2019-05-23	07:38	29.5	34.3	31	0.05	53.5	5.7	35.0	0.16
	K7	150	14.1	2019-05-25	06:06	29.4	34.4	18	0.03	58.2	4	50.1	0.08
	K8	155	11	2019-05-28	05:58	29.2	34.4	42	0.05	57.3	5.5	131.8	0.04
	K10	145	11	2019-06-02	05:19	29.6	34.3	32	0.05	51.6	8.1	76.5	0.10
	K12	135	11	2019-06-06	07:42	29.9	34.3	21	0.07	52.9	BLD	59.2	N/A
	K13	130	11	2019-06-07	05:46	29.9	34.2	35	0.06	42.6	4.6	35.5	0.13
	K14	127.92	15.5	2019-06-09	07:45	30.5	34.6	23	0.05	48.8	BLD	12.2	N/A
	K14a	124.37	19.12	2019-06-10	06:42	30.2	34.4	25	0.04	41.7	3.9	16.6	0.23
	**Mean**					**29.6 (0.5)**	**34.4 (0.2)**	**25.6 (8.0)**	**0.05 (0.01)**	**53.3 (2.7)**	**6.4 (3.1)**	**45.7 (32.5)**	**0.16 (0.11)**
NIO-SL	DT-01	79.50	6.49	2020-01-05	06:15	29.3	33	9	0.26	50.7	150	110	1.3
	DM2-2	80.96	5.50	2020-01-06	06:25	28.7	33.9	44	0.32	44.6	240	110	2.2
	SL1-2	82.20	6.50	2020-01-07	05:54	28.9	33.9	24	0.24	47.9	100	310	0.3
	**Mean**					**28.9 (0.2)**	**33.6 (0.4)**	**25.6 (14.3)**	**0.27 (0.03)**	**47.7 (2.5)**	**163 (58)**	**177 (94)**	**1.27 (0.77)**

*BLD represents that data value is below the detection limit (1 nmol L^–1^). N/A represents that data is not available. Bold values indicate mean values with standard deviation.*

Nutrient samples were collected in 100-ml high-density polyethylene (HDPE) bottles and were immediately measured onboard. Nanomolar levels of soluble reactive phosphorous were determined according to Ma et al. (2008) with a detection limit of 1.4 nM. Nanomolar levels of nitrate were analyzed using the chemiluminescent method (Garside, 1982) with a detection limit of 2 nM. Total Chl-a concentrations were determined from 300-ml seawater samples filtered onto 25-mm glass fiber filters (Whatman GF/F) under low vacuum (<0.02 MPa). Chl-a was extracted in 90% acetone and stored in darkness for 24 h under -20°C. The Chl-a concentration was determined fluorometrically using a pre-calibrated fluorometer (Turner Trilogy, United States) according to the method described by Welschmeyer (1994). For phytoplankton size structure measurement, we separated each sample to microphytoplankton (>10 μm), nanophytoplankton (3–10 μm), and picophytoplankton (0.7–3 μm) in these three groups based on size-fractionated chlorophyll a (Chl-a) measurements, according to standardized methods used in marine phytoplankton size research (Cermeño et al., 2006). The size-fractionated Chl-a concentration was determined using a 500-ml volume sample, which was filtered sequentially through 10- and 3-μm polycarbonate filters and 0.7-μm pore size GF/F filters. Filtering through 10 μm was done under gravity and for other pore sizes under low vacuum pressure (<0.02 MPa).

### *Trichodesmium* Colony Collection

To examine the variable chlorophyll fluorescence of natural *Trichodesmium*, samples were obtained with a 200-μm mesh, 1-m-diameter all-plastic net horizontally towed through surface layers when the ship was stationary. The net was kept a distance of 20 m away from the ship, and towing was conducted continuously for 15–20 min. After towing, *Trichodesmium* colonies were first poured into a 2–l HDPE bottle and then colonies were gently picked up with a micropipette. All colony samples were cleaned by washing three times with 0.2-μm filtered surface seawater in a 50-ml centrifugal tube to ensure that there were few other phytoplankton species in the samples. The colonies of *Trichodesmium* species were identified and counted using a 1-ml scaled slide and a Leica DM3000B microscope according to the trichome morphological characteristics (as per Jiang et al., 2018).

### Absorption Spectra of *Trichodesmium*

After washing with filtered seawater, approximately 15–20 colonies of *Trichodesmium* at four stations (K3, K14, DT-01, DM2-2) were gently filtered at the center of a 25-mm GF/F filter and kept under -80°C for further absorption spectra measurements. During laboratory processing, *Trichodesmium* absorption coefficients (*a*_*trico*_(), m^–1^) were determined using the quantitative filter technique of Cleveland and Weidemann (1993) as adapted by Wang et al. (2014) and Zhu et al. (2017).

### Photophysiology Measurements

Variable chlorophyll fluorescence was measured using a fast repetition rate fluorometer (FastOcean) integrated with a FastAct laboratory system (Act2, Chelsea Technologies Ltd, West Molesey, United Kingdom). The sample chamber of the instrument has three light-emitting diodes (LEDs) that provide flash excitation energy centered at 447 nm (blue), 519 nm (green), and 634 nm (orange). The 447-nm blue band corresponds to the absorption peak of Chl-a while 519 and 634 nm correspond to the absorption peaks of phycoerythrins and phycocyanins (Kazama et al., 2021). Two LED combinations (447 nm only and 447 + 519 + 634 nm) were used for FRRf measurements of all samples. Typically, triple *Trichodesmium* samples (each containing ca. 5–10 colonies) were measured after 30–60 min dark adaption, and the measurements for all three samples were completed within 1 h. FRRf measurements were corrected for blank fluorescence using 0.2-μm filtrates (Cullen and Davis, 2003). After dark acclimation, 5-ml subsamples with 5–10 *Trichodesmium* colonies were transferred into the FRRf. The instrument was programmed to deliver a single-turnover protocol with a saturation phase comprising 100 flashlets on a 2-μs pitch and a relaxation phase comprising 40 flashlets on a 60-μs pitch (as per Hoppe et al., 2015; Hughes et al., 2021). Each sample was exposed sequentially to one dark and nine actinic light levels (69, 158, 273, 421, 611, 621, 857, 1,173, and 1,580 mol quanta m^–2^ s^–1^) for a total of 1,000 s duration to retrieve a fluorescence-light response curve (FLC). A fluorescence transient was recorded from the average of one acquisition every 10 s (resulting in 10 acquisitions per light step). Each recorded transient was fitted to the biophysical model of Kolber et al. (1998) to determine the minimum fluorescence yield, maximum fluorescence yield, effective absorption, and photochemical efficiency of PSII for darkness (*F*_*o*_, *F*_*m*_, σ_*PSII*_, and *F*_*v*_/*F*_*m*_) and for each actinic light level (*F*′, $F_{m}^{\prime }$, $\sigma_{PSII}^{\prime }$, and $F_{q}^{\prime }/F_{m}^{\prime }$). The parameter τ_*ES*_ is the time constant of reoxidation of the primary stable electron acceptor *Q*_*A*_, and 1/τ_*ES*_ (ms^–1^) is an estimate of the rate of *Q*_*A*_ reoxidation. The normalized Stern–Volmer quenching coefficient (*NPQ*_*NSV*_, McKew et al., 2013) was calculated from these parameters as $F_{o}^{\prime }/F_{v}^{\prime }$ where $F_{\text{o}}^{\prime }$ was estimated as $F_{o}^{\prime }$ = *F*_*o*_*/(F*_*v*_/*F*_*m*_ *+*$F_{o}/F_{m}^{\prime }$*)* (Oxborough and Baker, 1997) and $F_{v}^{\prime }$ = ($F_{m}^{\prime }-F_{o}^{\prime })$*/*$F_{m}^{\prime }$. After dark acclimation, we calculated *NPQ*_*NSV*_ at first light step of zero PAR (*NPQ*_*NSV*_) and at saturation light (*E*_*K*_) level (*NPQ*_*NSV*_*Ek*_) during the light curve.

To account for the spectral differences between FRRF-LEDs and the natural light spectra *in situ*, we employed a σ_*PSII*_-correction factor (*F*) according to Eq. (11) following Suggett et al. (2006):


(1) $$F=\sigma_{PSII}^{abs}/\sigma_{PSII}^{FRR-LED}=\left(\frac{{\bar{a}}^{chl}\left(insitu\right)}{{\bar{a}}^{chl}\left(FRRf\right)}\right),$$

where $\sigma_{PSII}^{abs}$ represents spectral corrected σ_*PSII*_; ${\bar{a}}^{chl}\left(FRRf\right)$ and ${\bar{a}}^{chl}\left(insitu\right)$ represents the absorption coefficients weighted to the FRRf excitation spectra (either for blue-band or combination wavelengths) and *in situ* irradiance spectra, respectively. Detail calculations for ${\bar{a}}^{chl}\left(FRRf\right)$ and ${\bar{a}}^{chl}\left(\mathrm{in}\mathrm{situ}\right)$ can be found in Suggett et al. (2004) and Zhu et al. (2016).

The instantaneous PSII reaction center-normalized electron transport rate (ETR_*PSII*_, mol e^–^ [mol PSII]^–1^ s^–1^) for each light level was calculated as per Kolber and Falkowski (1993),


(2) $${\text{ETR}}_{PSII}=\text{PAR}\times \sigma_{PSII}\times F\times qP\times \Phi_{RC}\times 6.022\times 10^{-3}$$

where PAR is in units of μmol photons m^–2^ s^–1^ and σ_*PSII*_ is the effective absorption cross section of PSII (Å^2^ PSII^–1^). Φ_*RC*_ accounts for the assumption that one electron is produced from each RCII charge separation (Kolber and Falkowski, 1993), and the constant value 6.022×10^−3^ converts μmol quanta to quanta, PSII to mol PSII, and Å^2^ to m^2^ (Suggett et al., 2001). The term *q*_*p*_ (dimensionless) is the PSII operating efficiency and accounts for the extent of photochemical energy conversion by PSIIs, determined as ($F_{m}^{\prime }-F^{\prime }$)/($F_{m}^{\prime }-F_{o}^{\prime }$).

ETR_*PSII*_ and PAR data from the FRRf-light response curves were then fit to the photosynthesis-light dependency model of Platt et al. (1980):


(3) $$ETR_{PSII}=ETR_{PSII}^{max}\times \left(1-e^{-\alpha E/ETR_{PSII}^{max}}\right),$$

where $ETR_{PSII}^{max}$ is light-saturated ETR (mol e^–^ [mol PSII]^–1^ s^–1^), *E* is irradiance (μE m^–2^ s^–1^), and α = initial slope of the ETR-l curve (mol e^–^ mol RCII^–1^ s^–1^ (μmol quanta m^–2^ s^–1^)^–1^).

### Statistical Analyses

All correlations were examined using Spearman’s rank correlation coefficient. Kruskal–Wallis *t*-tests were applied for testing the significant differences between groups of data. All statistical analyses and curve fittings were performed using the open-source statistical software program R (version 3.6.1, R Core Team, 2019). Figures were plotted by Ocean Data View 5 (Schlitzer et al., 2018) and R software.

### Fast Repetition Rate Fluorometry-Derived Primary Productivity

We calculated Chl-a specific rates of primary production (*P^Chl^*, mgC mg Chl-a^–1^ h^–1^) from FRRf measurements in a similar fashion to Zhu et al. (2016, 2017) as follows:


$$P^{Chl}=\int_{t1}^{t2}ETR_{P\text{S}II}dt\times n_{PSII}\times 893\times 12÷\Phi_{e,C}$$

where $\int_{t1}^{t2}ETR_{P\text{S}II}dt$ denotes ETR_*PSII*_ scaled to hourly rates (where the period between *t1* and *t2* is 60 min), *n*_*PSII*_ is the number of PSII reaction centers per Chl-a (mol RCII [mol chl a] ^–1^) (see Suggett et al., 2010; Oxborough et al., 2012), _*e,C*_ is the electron requirement for carbon fixation (mol e^–^ per mol C) (see Lawrenz et al., 2013), the constant factor 893 converts mol Chl-a to mg Chl-a and mol e^–^ to mmol e^–^, and 12 converts mmol C to mgC. For this study, we used an assumed value of 0.027 mol RCII (mol Chl-a) ^–1^ for *Trichodesmium* as per Richier et al. (2012). Here we used previously reported experimental results (Boatman et al., 2018b, see their Figure 4D), to develop a simple light-dependent function describing _*e,C*_ for *Trichodesmium* in this study, where _*e,C*_ = 0.0115PAR + 5 (*n* = 24, *r*^2^ = 0.97)—we revisit the point, together with the choice of *n*_*PSII*_ in the discussion.

## Results

### Physical and Biochemical Environments

**FIGURE 2:**
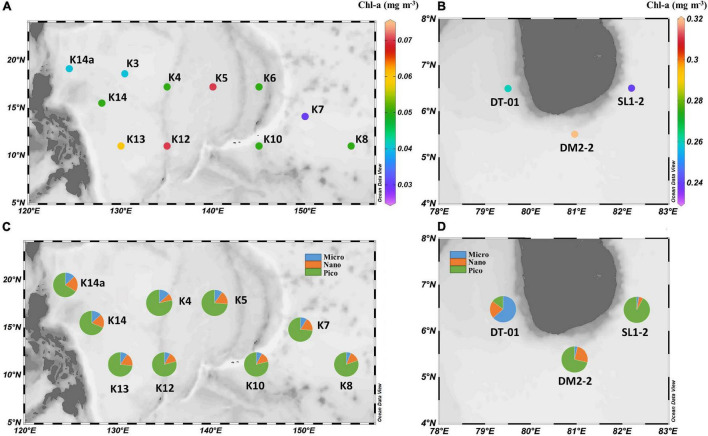
**(A,B)** Plots of measured Chlorophyll-a (Chl-a) concentration from the surface at each station. **(C,D)** Plots of surface microphytoplankton (> 10 μm), nanophytoplankton (3–10 μm), and picophytoplankton (0.7–3 μm) community fraction at each station.

Basic physical and biochemical parameters at sampling sites for two study regions are presented in Table 1. The mean value ± standard deviation (SD) of sea surface temperature (SST), surface salinity, MLD, and daily photosynthetically available radiation (PAR) at NWP were 29.6 ± 0.5°C, 34.4 ± 0.2, 25.6 ± 8.0 m, and 53.3 ± 2.7 mol photon m^–2^ d^–1^, respectively. Similar data ranges were observed for the NIO-SL area, with mean values of 28.9 ± 0.2°C, 33.6 ± 0.4, 25.6 ± 14.3 m, and 47.7 ± 2.5 mol photon m^–2^ d^–1^. Although there was no significant difference in silicate found between two areas (1.5 ± 0.1 vs. 2.0 ± 0.4 μmol l^–1^, *p* = 0.2), concentrations of NO_*x–*_ and PO_43–_ were significantly higher (Kruskal–Wallis *t*-test, *df* = 1, *p* < 0.05) for NIO-SL (163 ± 58 and 177 ± 94 nmol l^–1^) than for NWP (6.4 ± 3.1 and 45.7 ± 32.5 nmol l^–1^). The low N-to-P ratios (mean ± *SD*: 0.16 ± 0.11 for NWP and 1.27 ± 0.77 for NIO-SL) suggest severe N limitation of the overall phytoplankton community in both regions. Mean Chl-a (± SD) in the surface water of NWP was 0.05 (± 0.01) mg m^–3^, which was significantly lower than that of NIO-SL (0.27 ± 0.03 mg m^–3^; Kruskal–Wallis *t*-test, *df* = 1, *p* < 0.01; Figures 2A,B). Such low Chl-a in the NWP is typical of an oligotrophic ocean where nitrogen supply is insufficient (Dore et al., 2008). Microscopy revealed that *T. thiebautii* (likely belonging to Clade I, see Rouco et al., 2016) was the dominant *Trichodesmium* species in all surface samples taken from both NWP (Supplementary Figures 1A,B) and NIO-SL (Supplementary Figures 1C,D) regions. Size-fractionated Chl-a measurements revealed that picophytoplankton (< 3 μm) dominated in NWP surface samples, with an average fraction of 75 ± 5% (Figure 2C). In the NIO-SL area, picophytoplankton dominated at both DM2-2 and SL1-2, with an average fraction of 81% (Figure 2D). However, DT-01 was dominated by micro-phytoplankton (> 10 μm, 63.6%). Flow cytometry data further confirmed that *Prochlorococcus* accounted for 96% and 83% of the picophytoplankton group at NWP and NIO-SL, respectively (Supplementary Table 1).

### Photophysiology of *Trichodesmium* at Northwest Pacific and North Indian Ocean in the Vicinity of Sri Lanka

**FIGURE 3:**
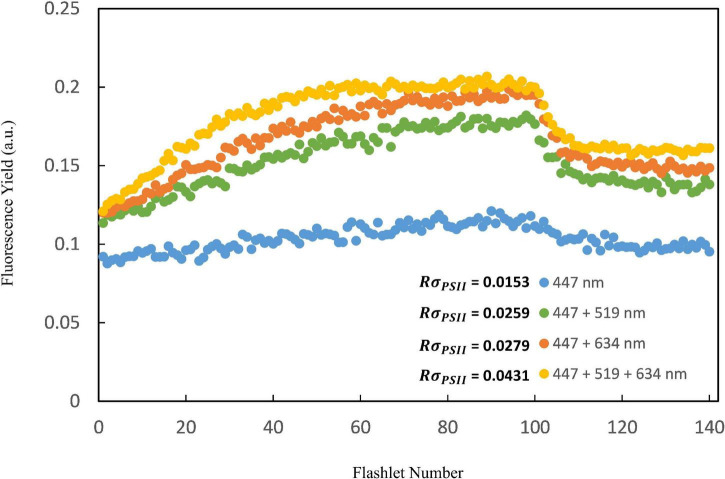
The raw fast repetition rate (FRR) flurometer fluorescence induction kinetic curves of four different lights combinations (blue: 447 nm; blue + green: 447 + 519 nm; blue + orange: 447 + 634 nm and blue + green + orange: 447 + 519 + 634 nm) for the *Trichodesmium* study (using K14 as an example). Results showed that the FRRf measurement protocol consisting of three excitation wavelengths combined generated the highest quality data (*R*σ_*PSII*_ > 0.03).

The performance of the multi-excitation FRR fluorometer for *Trichodesmium* measurements was first assessed by comparing photophysiological results between the multiple-wavelength combination (i.e., 447 + 519 + 634 nm combined) and that of the sole blue-excitation LED (447 nm) generated from single-turnover fluorescence induction. In general, very flat induction curves were observed for natural *Trichodesmium* when blue light only was used as the fluorescence excitation flash (Figure 3), indicating that the blue LED was insufficient to drive complete PSII reaction center closure. Indeed, as shown in Table 2, in NWP the mean value of *F*_*v*_/*F*_*m*_ derived from blue-band excitation was 60% of the value from excitation combination of three bands (0.31 ± 0.08 vs. 0.51 ± 0.07, Kruskal–Wallis *t*-test, *p* < 0.05). In NWP, a significantly lower value of *NPQ*_*NSV*_ was observed in excitation by the combined LED protocol compared to the blue LED only (1.49 ± 0.36 vs. 2.92 ± 0.99, Kruskal–Wallis test, *p* < 0.01). Lower average σ_*PSII*_ was found for blue-band excitation compared to the value for combination band excitation after spectral correction (67 ± 17 vs. 79 ± 28 Å^2^ PSII^–1^, Kruskal–Wallis *t*-test, *p* = 0.18). Although similar trends were visually observed in the dataset of NIO-SL *Trichodesmium*, no statistical differences were found, likely due to the limited data (Kruskal–Wallis test, *p* > 0.05). The FRRf data quality with each excitation combination was assessed by the probability of an RCII being closed during the first flashlet of a single turnover saturation phase under dark conditions (*R*σ_*PSII*_), and low-quality data were frequently observed when PSII was excited with 447 nm (here we define low-quality data as corresponding to an *R*σ_*PSII*_< 0.03; Figure 3). Meanwhile, at most stations, the ETR-light response could not be successfully fitted from FRRf measurement when only the blue band was used for the excitation flash (Table 2). As such, only results from combined measuring protocol (447 + 519 + 634 nm) were analyzed further.

**TABLE 2:** Comparison of mean (SD) of photophysiology measurement results of *F*_*v*_/*F*_*m*_, σ_*PSII*_ (Å^2^ PSII^–1^), NPQ at dark (*NPQ*_*NSV*_), 1/τ_*ES*_ (ms^–1^), *a*_*ETR*_ (mol e^–^ mol RCII^–1^ s^–1^ (μmol quanta m^–2^ s^–1^)^–1^), *ETR*_*max*_ (mol e^–^ [mol PSII]^–1^ s^–1^), and *E*_*k*_ (mol quanta m^–2^ s^–1^) between exciting PSII with 447 nm and with combination band (447 + 519 + 634 nm) in the NWP and NIO-SL.

Region	λ_*ex*(*nm*)_	*F_v_/F_m_*	*σ_PSII_*	*NPQ_NSV_*	1/τ_*ES*_	*a_ETR_*	*ETR_max_*	*E_k_*
NWP	447	0.31 (0.08)	67 (17)	2.92 (0.99)	0.33 (0.05)	N/A	N/A	N/A
	447 + 519 + 634	0.51 (0.07)	79 (28)	1.49 (0.36)	0.41 (0.11)	0.52 (0.18)	388 (185)	846 (472)
NIO-SL	447	0.18 (0.08)	145 (55)	7.47 (3.98)	0.31 (0.15)	N/A	N/A	N/A
	447 + 519 + 634	0.29 (0.05)	140 (8)	3.17 (1.02)	0.41 (0.04)	0.67 (0.35)	374 (272)	524 (136)

*N/A represents that data is not available.*

**FIGURE 4:**
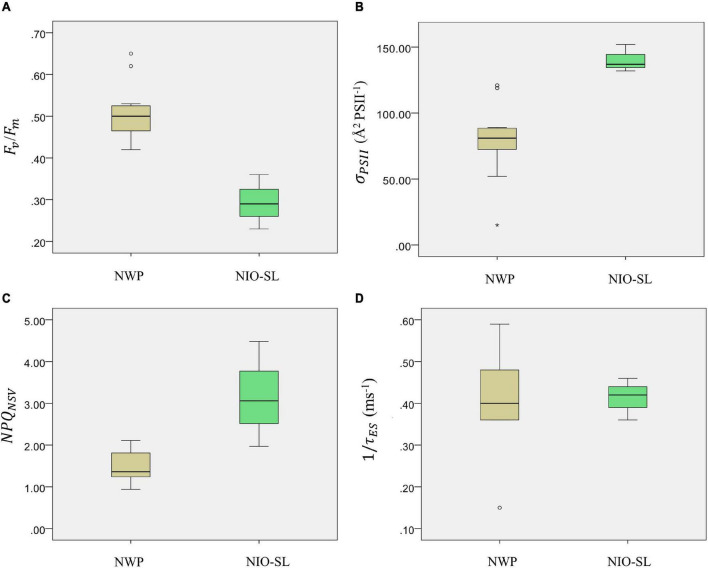
Boxplots of *Trichodesmium* photophysiological parameters **(A)** PSII maximum photochemical efficiency, *F*_*v*_/*F*_*m*_, **(B)** functional absorption cross section of PSII, σ_*PSII*_ (Å^2^ PSII^–1^), **(C)** normalized Stern–Volmer non-photochemical quenching *NPQ*_*NSV*_ and **(D)** PSII turnover time 1/τ_*ES*_ (ms^–1^) within NWP and NIO-SL regions. * indicates the extreme value.

FRRf-derived photophysiological parameters for *Trichodesmium* sp. samples collected from the two study areas were compared (see Table 3 and Figure 4). Except for 1/τ_*ES*_, significant differences in *F*_*v*_/*F*_*m*_, σ_*PSII*_, and *NPQ*_*NSV*_ were found between NWP and NIO-SL. Specifically, after blank correcting (blank values of *F*_*o*_ and *F*_*m*_ were in the range of 0.02–0.05), the *F*_*v*_/*F*_*m*_ of *Trichodesmium* in the NWP ranged from 0.42 at station K6 to a maximum of 0.65 at station K13, with a mean value of 0.51 ± 0.07. Significantly lower *F*_*v*_/*F*_*m*_ values were observed for NIO-SL data compared to NWP and ranged from 0.23 at DM2-2 to 0.36 at DT-01 with an average of 0.29 ± 0.05 (Kruskal–Wallis *t*-test, *df* = 1, *p* = 0.01; Figure 4A). Effective absorption cross sections, σ_*PSII*_, of *Trichodesmium* varied from 15 at K10 to 121 Å^2^ PSII^–1^, with a mean of 79 ± 28 Å^2^ PSII^–1^. This value was only approximately half of the mean σ_*PSII*_ of *Trichodesmium* in the NIO-SL (140 ± 8, ranged from 132 to 152 Å^2^ PSII^–1^; Figure 4B, Kruskal–Wallis test, *df* = 1, *p* = 0.01). *NPQ*_*NSV*_ was ca. twofold higher in the NIO-SL (3.17 ± 1.0) than in the NWP *Trichodesmium* samples (1.49 ± 0.36; Kruskal–Wallis *t*-test, *df* = 1, *p* = 0.024; Figure 4C). The smallest value of *NPQ*_*NSV*_ was observed at K13 in the NWP and at DT-01 in the NIO-SL (Table 3). Meanwhile, analysis showed that mean values of *NPQ*_*NSV*_*Ek*_ were higher than *NPQ*_*NSV*_ in both NWP and NIO-SL regions (Table 3).

**TABLE 3:** Value of photophysiological parameters, *F*_*o*_ (arbitrary units: a.u.), *F*_*v*_/*F*_*m*_, σ_*PSII*_ (Å^2^ PSII^–1^), NPQ at dark (*NPQ*_*NSV*_), NPQ at *E*_*k*_ light (*NPQ*_*NSV*_*Ek*_), and 1/τ_*ES*_ (ms^–1^) at each sampling station measured by FRRf with combination band.

Region	Stn.	*F_o_*	*F_v_/F_m_*	*σ_PSII_*	*NPQ_NSV_*	*NPQ_NSV_Ek_*	1/τ_*ES*_
NWP	K3	0.30	0.46	119	1.35	1.42	0.45
	K4	0.22	0.52	72	1.19	0.97	0.36
	K5	0.36	0.52	81	1.08	2.08	0.36
	K6	0.4	0.42	77	1.98	1.60	0.36
	K7	0.33	0.48	81	1.29	1.94	0.56
	K8	0.13	0.50	52	1.86	2.18	0.36
	K10	0.03	0.62	15	2.11	N/A	0.15
	K12	0.11	0.47	89	1.36	1.58	0.40
	K13	0.10	0.65	121	0.94	1.73	0.48
	K14	0.11	0.53	73	1.48	1.65	0.48
	K14a	0.08	0.42	88	1.76	1.58	0.59
	**Mean**	**0.19 (0.12)**	**0.51 (0.07)**	**79 (28)**	**1.49 (0.36)**	**1.67 (0.33)**	**0.41 (0.11)**
NIO-SL	DT-01	1.9	0.36	137	1.97	1.96	0.42
	DM2-2	0.62	0.23	152	4.48	5.46	0.36
	SL1-2	1.1	0.29	132	3.06	7.1	0.46
	**Mean**	**1.2 (0.5)**	**0.29 (0.05)**	**140 (8)**	**3.17 (1.0)**	**4.84 (2.14)**	**0.41 (0.04)**
*P*		**0.01**	**0.01**	**0.01**	**0.024**	**0.01**	0.9

*Regional mean value (SD) was presented in bold. Kruskal–Wallis t-tests results are shown comparing the difference between the regions. Values in bold indicate significant differences where p < 0.05.*

Derived from ETR-I curves, the initial slope α_*ETR*_, maximum electron turnover rate $ETR_{PSII}^{max}$, and light saturation of PSII charge separation *E*_*K*_ (= $ETR_{PSII}^{max}$/α_*ETR*_, mol quanta m^–2^ s^–1^) of *Trichodesmium* in the two study areas are presented in Table 4. According to the results, a lower average value of α_*ETR*_ and a higher $ETR_{PSII}^{max}$ were observed for *Trichodesmium* in the NWP than in the NIO-SL, resulting in a higher averaged *E*_*K*_ of *Trichodesmium* in the NWP. However, a Kruskal–Wallis test showed that there were no significant differences in these three variables between the NWP and NIO-SL (*df* = 1, *p* = 0.39). The values observed at K13 and DT-01 were quite different from the data of other stations (Table 4), which will be discussed further below. FRRf-retrieved rates of primary productivity (*P^Chl^*) ranged from 7.8 to 21.1 mgC mg Chl-a^–1^ h^–1^ with a mean value of 11.9 ± 4.4 mgC mg Chl-a^–1^ h^–1^ (Table 5).

**TABLE 4:** Value of α_*ETR*_ [mol e^–^ mol RCII^–1^ s^–1^ (μmol quanta m^–2^ s^–1^)^–1^], $ETR_{PSII}^{max}$ [mol e^–^ [mol PSII]^–1^ s^–1^], and *E*_*K*_ (mol quanta m^–2^ s^–1^) at each sampling station derived from ETR-light curves.

Region	Stn.	*α_ETR_*	${\text{ETR}}_{\mathrm{PSII}}^{\max}$	*E_K_*
	K3	0.24	366	1,525
NWP	K4	0.37	393	1,062
	K5	0.53	169	319
	K6	0.5	849	1,698
	K7	0.6	219	365
	K8	0.42	352	838
	K12	0.58	264	455
	K13	0.96	282	293
	K14	0.53	529	998
	K14a	0.5	455	910
	**Mean**	**0.52 (0.18)**	**388 (185)**	**846 (472)**
NIO-SL	DT-01	1.13	757	670
	DM2-2	0.62	213	341
	SL1-2	0.27	151	560
	**Mean**	**0.67 (0.35)**	**374 (272)**	**524 (136)**
*P*		0.39	0.39	0.39

*Regional mean value (SD) was presented in bold. Kruskal–Wallis t-tests results are shown comparing the difference between the regions.*

**TABLE 5:** Hourly Chl-a specific *ETRs* (mmol e^–^ mg Chl-a^–1^ h^–1^), estimates of electron requirement for carbon fixation (_*e,C*_, mol C per mol e^–^) and primary productivity of *Trichodesmium* (mgC mg Chl-a^–1^ h^–1^) for seven stations in the morning (07:00 a.m.).

Stn.	*ETR_hour_*	Φ_**e*,*C**_	*P^Chl^*
K3	5.5	7.6	8.7
K5	5.9	6.4	11.0
K8	4.7	6.2	9.0
K13	12	6.8	21.1
K14a	5.4	6.2	10.4
DT-01	7.4	5.7	15.4
DM2-2	3.7	5.7	7.8
**Mean**	**6.3 (2.5)**	**6.4 (0.6)**	**11.9 (4.4)**

*Bold values indicate mean values with standard deviation.*

### Correlation Analyses Within Photophysiological Factors and Between Photophysiological and Environmental Data

**FIGURE 5:**
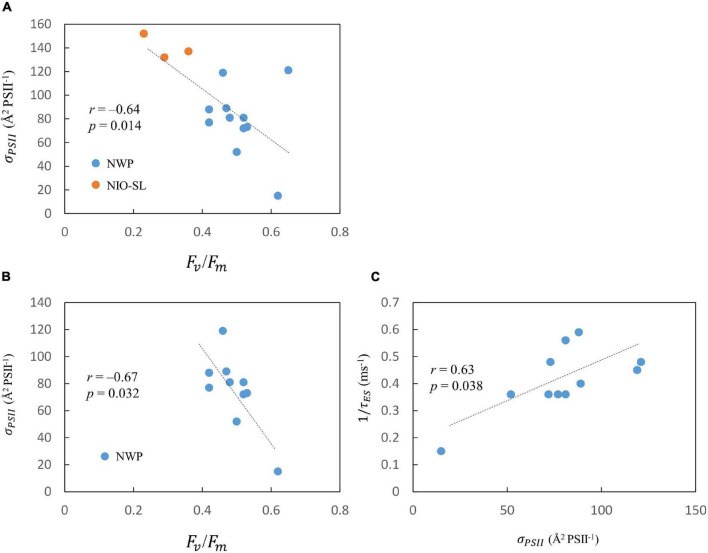
Scatter plots of **(A)** PSII maximum photochemical efficiency, *F*_*v*_/*F*_*m*_, and functional absorption cross section of PSII, σ_*PSII*_ (Å^2^ PSII^–1^) for pooled data. Scatter plots of **(B)** *F_v_*/*F*_*m*_ and σ_*PSII*_ **(C)** σ_*PSII*_ and PSII turnover time 1/τ_*ES*_ for NWP data only.

After pooling all data, correlations within FRRf-derived photophysiological factors were examined by Spearman analyses. We found that *F*_*v*_/*F*_*m*_ negatively correlated with both σ_*PSII*_ (*n* = 14, *r* = –0.64, *p* = 0.014; Figure 5A). When K13 was excluded, an inverse relationship between σ_*PSII*_ and *F*_*v*_/*F*_*m*_ was observed for the NWP dataset as well (*n* = 10, *r* = –0.67, *p* = 0.032; Figure 5B). A strong positive relationship was found between σ_*PSII*_ and 1/τ_*ES*_ in the NWP dataset (*n* = 11, *r* = 0.63, *p* = 0.038, Figure 5C). The same analyses were then conducted to examine the correlation of ETR-E curve-derived α_*ETR*_ and $ETR_{PSII}^{max}$, but no significant correlation was found (*p* = 0.9).

The Spearman rank correlation coefficients between the photophysiology of *Trichodesmium* and environmental factors are presented in Table 6. *F*_*v*_/*F*_*m*_ was positively correlated with temperature (*n* = 14, *r* = 0.546, *p* = 0.043) and light intensity (*n* = 14, *r* = 0.601, *p* = 0.023) but negatively correlated with the N-to-P ratio (*n* = 10, *r* = -0.632, *p* = 0.014). σ_*PSII*_ was positively correlated with Chl-a (*n* = 14, *r* = 0.563, *p* = 0.036) and the N-to-P ratio (*n* = 10, *r* = 0.772, *p* = 0.009). Nutrients are likely to affect the variations in *NPQ*_*NSV*_. Both NO_2_ + NO_3–_ and PO_43–_ had positive correlations with *NPQ*_*NSV*_ (*n* = 10, *r* = 0.648, *p* = 0.043 and *r* = 0.565, *p* = 0.035). For ETR-I curve-derived parameters, *E*_*K*_ was found to be positively related to salinity (*n* = 13, *r* = 0.6, *p* = 0.03). Although significant correlations were observed between environmental and photophysiological factors, it is not likely that temperature, salinity, and light intensity are the controlling factors in this study. For example, in fact, *F*_*v*_/*F*_*m*_ is largely independent of temperature (Geider et al., 1993) and a decrease in *F*_*v*_/*F*_*m*_ is typically observed in response to increased light intensity due to the NPQ (Suggett et al., 2010). It is thus important to explore the main factors determining the variability of phytoplankton photophysiology, which will be discussed in the next section.

**TABLE 6:** Spearman correlation coefficients for correlations between photophysiological parameters and environmental variables.

Parameters	Temp	Chl-a	PAR	NO_2_+NO_3–_	PO_43–_	N/P
*F*_*v*_/*F*_*m*_	**0.546** 0.043 *n* = 14		**0.601** 0.023 *n* = 14			−**0.632** 0.014 *n* = 10
σ_*PSII*_		**0.563** 0.036 *n* = 14				**0.772** 0.009 *n* = 10
*NPQ*_*NSV*_				**0.648** 0.043 *n* = 10	**0.565** 0.035 *n* = 10	

*Only significant correlation (bold values) between two variables (p < 0.05) was presented.*

## Discussion

### Application of Multi-Excitation Wavelength Fast Repetition Rate Fluorometry Study on Natural *Trichodesmium*

**FIGURE 6:**
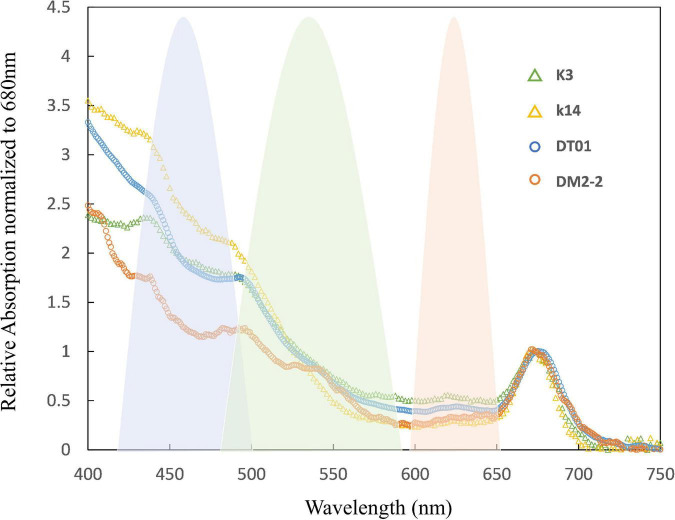
Normalized spectral absorption characteristics of *Trichodesmium* measured at four sampling stations (K3, K14, DT-01, DM2-2). The blue, green, and orange shades are the excitation spectrum (relative to quanta) of the light source of the FastAct2 (see “Materials and Methods” section).

Eukaryotic phytoplankton possessing pigment–protein complexes with chlorophylls and carotenoids usually exhibit efficient PSII absorption of FRR blue excitation. However, in cyanobacteria, the principal light-harvesting complexes are phycobilisomes, which have absorption peaks centered at 495, 545, and 565 nm (Fujita and Shimura, 1974). The latter two peaks correspond to phycoerythrobilin (PEB) while the first corresponds to phycourobilin (PUB). Suggett et al. (2009b) reported that the PSII action spectrum for cyanobacteria is typically much lower in blue light than in orange light. Raateoja et al. (2004) proposed that FRRf with an excitation light around 475 nm (targeting Chl-a) is not well-suited to studies of cyanobacteria *Nodularia spumigena* and *Aphanizomenon* sp., because their effective absorption is restricted to wavelengths beyond 550 nm. However, this is likely not the case for *Trichodesmium* because this bacterium contains a higher concentration of PUB than PEB (i.e., the cells absorb shorter wavelengths of light). This hypothesis is supported by our absorption spectrum of *Trichodesmium*, which showed greater absorption at shorter wavelengths in both NWP and NIO-SL samples (Figure 6). This conclusion has also been presented in previous studies (e.g., Subramaniam et al., 1999). Indeed, the relatively higher and lower absorption of PUB found for K14 and DM2-2 samples, respectively, corresponded to their notably higher and lower values of *F*_*v*_/*F*_*m*_ (0.53 and 0.23, Table 3). Cai et al. (2015) reported that *Trichodesmium* cells grown under high light showed a higher peak ratio of PUB to PEB, implying increased photoprotection associated with increased PUB (Subramaniam et al., 1999). Observed ratios of PUB to PEB in our data ranged from 1.6 in DM2-2 to 3.4 for K14. This appeared consistent with the higher *E*_*K*_ value measured for K14 (998 mol quanta m^–2^ s^–1^) compared to that of DM2-2 (341 mol quanta m^–2^ s^–1^, Table 4), suggesting that *Trichodesmium* were acclimated to higher irradiance in the NWP region. Using FRRf excitation at 447 nm (blue light), we observed low photosynthetic activity of *Trichodesmium* sampled from both areas (i.e., low *F*_*v*_/*F*_*m*_). These results suggest that the blue light (<500 nm) absorbed by *Trichodesmium* antenna pigments does not make a major contribution to the reduction of *Q*_*A*_ and the PQ pool, or to O_2_ evolution (Kazama et al., 2021). Subramaniam et al. (1999) suggested that under high light conditions, energy absorbed by PUB at 495 nm does not reach PSII and a large fraction is emitted as fluorescence at 565 nm. The O_2_ evolution driven by PEB absorption around 550 nm remains largely unaffected, and the energy absorbed by PEB is efficiently transferred to PSII. In our measurements, although there is a significant increase in *R*s_*PII*_ when either the 519- or 634-nm LEDs are added to the 447-nm LED, it is only when all three wavelengths are used together that *R*s_*PII*_ falls within the optimum range of 0.04–0.05 (K14 data as example, Figure 2). This most likely reflects a poor match between the fluorescence excitation spectrum and the output from the 447-nm LEDs, and a less than perfect match with the 519- and 634-nm LEDs (Oxborough, 2013). Our results provide the first evidence that combining green and orange bands of longer wavelengths as fluorescence excitation is the key to efficient PSII light harvesting and accurate estimation of the physiological responses of *Trichodesmium*.

### Photophysiology Parameters Vary in *Trichodesmium* Under Different Environmental Conditions

The FRRf-derived parameter *F*_*v*_/*F*_*m*_ represents an estimate of the maximum quantum yield of photochemistry (Kolber and Falkowski, 1993) and usually is considered as a reliable indicator for phytoplankton stress (Kolber et al., 1990; Geider et al., 1993). In the NWP, we observed a relatively high average *F*_*v*_/*F*_*m*_ of 0.51 ± 0.07. This value is very close to the mean value of 0.5 from laboratory-cultured *Trichodesmium* grown at 30°C (Breitbarth et al., 2007; [PhytoPAM, blue–green–red LED]) and slightly higher than values for natural populations observed in the North Atlantic by blue LED only (0.37–0.47) (Richier et al., 2012; [FIRe, blue LED]). However, it is important to note that *F*_*v*_/*F*_*m*_ varies not only among LED colors but also instruments that follow different measurement protocols. For example, higher values of *F*_*v*_/*F*_*m*_ are observed using instruments where the saturation protocol induces multiple turnover (MT) of PSII (e.g., PAM, Schreiber et al., 1986) compared to single turnover (ST) such as the FRRf in this study (Kromkamp and Forster, 2003).

When comparing results measured with the same type of fluorometer, the *F*_*v*_/*F*_*m*_ of *Trichodesmium* measured here are higher than other cyanobacteria, which typically range from 0.1 to 0.4 (Suggett et al., 2009b; FRRf, [blue LED]), although values larger than 0.6 have been observed for some species of nitrogen fixing diazotrophic cyanobacteria (Berman-Frank et al., 2007; FRRf, [blue-green LED]). We observed significantly lower *F*_*v*_/*F*_*m*_ of *Trichodesmium* in the NIO-SL (0.29 ± 0.05) compared to the value for *Trichodesmium* in the NWP (*p* = 0.01, Table 3), although the nutrient level was higher in the NIO-SL region (Table 1). *Trichodesmium* are not N-limited as they fix atmospheric nitrogen and provide this to N-limited ecosystems. However, because there is a theoretically unlimited supply of atmospheric N_2_, *Trichodesmium* often becomes phosphorus (P) limited (Wu et al., 2000; Hynes et al., 2009; Frischkorn et al., 2018). Meanwhile, because the nitrogenase enzyme is Fe-demanding and PSI:PSII ratios are elevated, *Trichodesmium* also routinely experience iron limitation (Berman-Frank et al., 2001; Chappell et al., 2012; Walworth et al., 2016). We observed that PO_43–_ was about fourfold higher in the NIO-SL (177 ± 94 nmol l^–1^) than in the NWP (45.7 ± 32.5 nmol l^–1^), and a low dissolved Fe concentration (DFe) was found in the NWP area, ranging from 0.09 to 0.26 with a mean value of 0.12 ± 0.05 nmol l^–1^ (Supplementary Figure 2; see also Browning et al., 2021 describing the same study area). Although we did not measure DFe in the NIO-SL, Twining et al. (2019) reported a concentration of ca. 0.2 nmol l^–1^ of DFe at the surface of Bay of Bengal and North Indian Ocean, which is similar to the DFe value of NWP. Thus, while Fe stress could prevail in both NWP and NIO-SL surface waters, the higher *F*_*v*_/*F*_*m*_ in the NWP is likely to suggest that rapid Fe recycling exists there (Kolber et al., 1990; Geider et al., 1993), which is favorable for *Trichodesmium* growth (Mills et al., 2004; Moore et al., 2008). A significantly higher mean value of *F*_*o*_ was observed for *Trichodesmium* in the NIO-SL (1.2 ± 0.5) compared to the NWP (0.19 ± 0.12, Table 3), where increased *F*_*o*_ decreases variable fluorescence (*F*_*v*_) and subsequently *F*_*v*_/*F*_*m*_. It is likely most of the variability in *F*_*o*_ is linked to changes in overall chlorophyll-a concentrations (with higher Chl-a increasing *F*_*o*_; Mean Chl in NIO-SL / NWP = 5.4; Mean *F*_*o*_ in NIO-SL/NWP = 6.3, Tables 1, 3). However, phycobilipigment fluorescence can also elevate *F*_*o*_ in the PSII Chla fluorescence band and thus reduce *F*_*v*_/*F*_*m*_ (Campbell et al., 1998). This phycobiliprotein contribution to *F*_*o*_ fluorescence could reflect low-yield fluorescence emission from coupled phycobilisomes or a high-yield emission from a small population of uncoupled phycobilisomes, which is related to their mobility in thylakoid membranes (Mullineaux et al., 1997).

A significant difference was also found in σ_*PSII*_of *Trichodesmium* collected from the two regions. A lower average value was observed in the NWP (79 ± 28 Å^2^ PSII^–1^) than in the NIO-SL (140 ± 8 Å^2^ PSII^–1^; *p* = 0.01, Table 3). Both values are within the reported range of ∼35–180 Å^2^ PSII^–1^ of laboratory-cultured *Trichodesmium erythraeum* IMS101 (Cai et al., 2015; [blue-green LED]; Boatman et al., 2018a,b; [blue LED]). σ_*PSII*_ broadly shows an inverse relationship with *F*_*v*_/*F*_*m*_ when all *Trichodesmium* are pooled together (Figure 4A). Phytoplankton increasing σ_*PSII*_ with decreasing *F*_*v*_/*F*_*m*_ is considered as a response to decreased growth irradiance, cell size, and increased physiological stress (Kolber et al., 1988; Moore et al., 2005, 2006; Suggett et al., 2009b). While cell size is not the main driver of variation in our surface *Trichodesmium* study, higher *E*_*k*_ was observed for *Trichodesmium* in the NWP than in the NIO-SL (Table 4), which is probably reflective of acclimation to higher irradiance for surface *Trichodesmium* at NWP than it at surface water of NIO-SL. Thus, the difference of in growth irradiance of *Trichodesmium* at two regions can be one explanation for the inverse relationship of *F*_*v*_/*F*_*m*_ and σ_*PSII*_ for our *Trichodesmium* dataset.

Lower *F*_*v*_/*F*_*m*_ and higher σ_*PSII*_ may also be associated with Fe stress (Behrenfeld and Milligan, 2013; Browning et al., 2014a). Under Fe-limited conditions, photoinactivated PSII reaction centers and/or an excess pool of partially energetically disconnected light-harvesting complexes accumulate within the thylakoid membrane, which could account for the lower values of *F*_*v*_/*F*_*m*_ (Behrenfeld and Milligan, 2013). Meanwhile, less efficient connectivity between active and photoinacivated PSIIs and/or a higher light-harvesting complex : reaction center ratio could account for the increase in σ_*PSII*_ (Boatman et al., 2018a). In addition, under Fe limitation, nitrogenase activity decreases, thus diminishing a major sink for reductant and energy that is otherwise supplied by respiratory electron flow through the Cyt *b_6_f* complex (Boatman et al., 2018a). This consequently restricts electron transport from PSII to the Cyt *b_6_f* complex, resulting in a decrease in electron transfer rate (1/τ, Timmermans et al., 2001; Hopkinson et al., 2007). Meanwhile, this potential electron bottleneck at the Cyt *b_6_f* complex would be expected to cause a strong plastoquinone (PQ) pool reduction, which consequently derives changes in both *F*_*v*_/*F*_*m*_ and σ_*PSII*_ (see Behrenfeld and Milligan, 2013).

**FIGURE 7:**
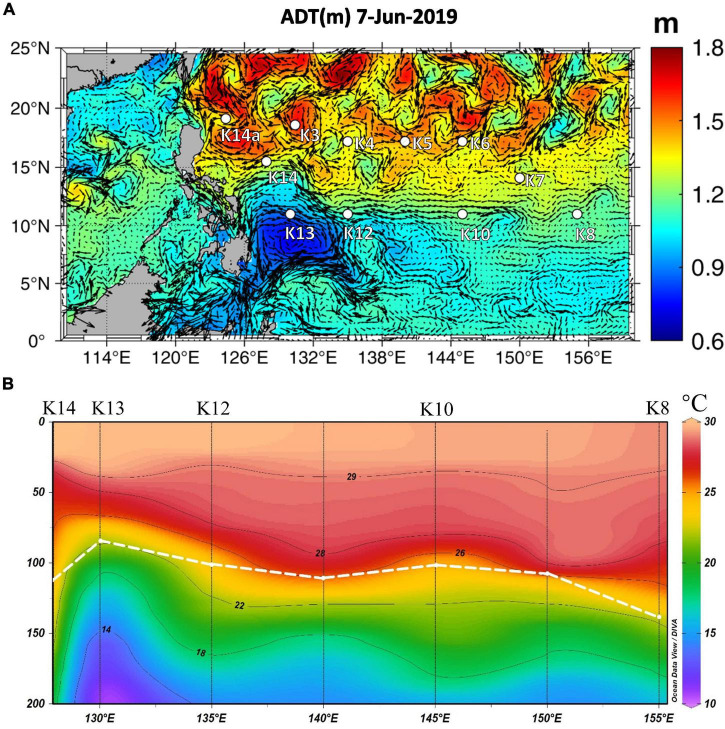
**(A)** Absolute dynamic topography (ADT) data for time of sampling at station K13 (data downloaded from https://resources.marine.copernicus.eu/). **(B)** Temperature profile of section from K8 to K14, highlighting upwelling observed at station K13. White dotted line indicates mixed layer depth.

In addition to the variation in physiological parameters that was observed when comparing different areas, data variation within the same region also suggests that *Trichodesmium* responds favorably to increased nutrients (i.e., Fe and P). As shown in the “Results” section, the highest values of *F*_*v*_/*F*_*m*_ (0.65) and σ_*PSII*_ (121 Å^2^ PSII^–1^) were found at K13 in the NWP within the Mindanao eddy upwelling zone, which is characterized by its cold anomaly at ∼100 m depth east of Mindanao (Udarbe-Walker and Villanoy, 2001; Figure 7A). Although the surface P and Fe concentrations at K13 did not differ significantly from other stations (Table 1 and Supplementary Figure 2), nutrient pumping from deeper depths likely supported the phytoplankton growth as seen in the shallowing of DCM (∼85 m) at the upwelling station K13 compared to the nearby stations (∼100 m, Figure 7B; Falkowski et al., 1991). However, both highest values of *F*_*v*_/*F*_*m*_ and σ_*PSII*_ being observed together at K13 are not what would be expected as usually an increase in *F*_*v*_/*F*_*m*_ follows a decrease in σ_*PSII*_ (Suggett et al., 2009b). A similar result was observed during IronEx II when Behrenfeld et al. (1996) reported that within 24 h of Fe addition, the *F*_*v*_/*F*_*m*_ and σ_*PSII*_ of prokaryotic-dominated communities increased by a factor of two and four, respectively. Behrenfeld et al. (2006) interpreted these physiological changes as indicative of a re-coupling to PSII of detached iron-stress-induced antenna proteins, resulting in a simultaneous increase of σ_*PSII*_ and lower values of *F*_*o*_ and *F*_*m*_ that resulted in an increase in *F*_*v*_/*F*_*m*_. Such a response may be specific to relieving the Fe limitation of prokaryotes (Suggett et al., 2009b). In the NIO-SL dataset, the maximum *F*_*v*_/*F*_*m*_ of *Trichodesmium* was found at station DT-01. As with station K13 in the NWP, no significant difference in surface PO_43–_ was found between DT-01 and the other stations (Table 1; but the difference in DFe is unknown). However, the large-size phytoplankton (> 10 μm) dominating at DT-01 (Figure 2D) probably suggests a less nutrient stressed condition, as large phytoplankton often dominated at higher nutrient waters (Ciotti et al., 2002; Cermeño et al., 2006; Roy et al., 2013). Therefore, increased *F*_*v*_/*F*_*m*_ responding to the alleviation of Fe and P limitation in both NWP and NIO-SL surface water also indicates that at local scales, Fe and P availability may have considerable effects on the variability in PSII characteristics of *Trichodesmium*.

Non-photochemical fluorescence quenching (NPQ) acts to dissipate excitation energy and hence protect RCII from damage (Krause and Weis, 1991; Horton et al., 1996; Alderkamp et al., 2010; Croteau et al., 2021). A tight relationship between NPQ and light intensity has been widely reported (Olaizola et al., 1994; Campbell et al., 1998; Papageorgiou et al., 2007; Ihnken et al., 2011; Schuback et al., 2015). NPQ formation is important for *Trichodesmium* because these N-fixing cyanobacteria form blooms at the surface of nutrient-poor tropical and subtropical oceans and thus require adequate photoprotection in such high light environments (Gorbunov et al., 2011). We observed higher mean values of *NPQ*_*NSV*_ for *Trichodesmium* in the NIO-SL (3.17) than in the NWP (1.49). Increased NPQ may result from quenching within a proportion of the closed RCIIs (Krause and Weis, 1991). However, the higher NPQ observed for *Trichodesmium* in the NIO-SL does not correspond to smaller σ_*PSII*_ but rather to larger values, suggesting a different mechanism to explain the higher NPQ in the NIO-SL. Nutrients affect PSII variable fluorescence and photoinhibition in natural phytoplankton communities, which could affect NPQ as well. Kulk et al. (2013) reported a significant NPQ decrease during nutrient starvation in *Prochlorococcus marinus*. In addition, studies have found that elevated Fe stress possibly increases NPQ, which could result from a “bottleneck effect” for electron transport before photosystem I (Browning et al., 2014b; Schuback et al., 2015; Ryan-Keogh and Thomalla, 2020). Thus, we speculate that the higher *NPQ*_*NSV*_ found for *Trichodesmium* in the NIO-SL may also result from *Trichodesmium* growing under more Fe-stressed conditions. When we excluded the difference in growth states and focused only on NWP data, a decrease in σ_*PSII*_ in response to the increase in NPQ was found (Spearman, *n* = 11, *r* = –0.565, *p* = 0.07). This observation agrees well with numerous previous studies that similarly report the existence of a negative correlation between these two parameters (Ihnken et al., 2011; Lavaud et al., 2016). An increase in NPQ with a decrease in σ_*PSII*_ is indicative of NPQ occurring in the PSII light-harvesting antennae, whereby reducing the flux of absorbed energy from phycobilisomes to reaction centers leads to a corresponding decrease in σ_*PSII*_ (Gorbunov et al., 2011).

The initial slope parameter, α_*ETR*_, and maximum electron turnover rate, $ETR_{PSII}^{max}$, were both derived from ETR-light response curves, describing the light-limited and light-saturated rate of electron transport, respectively (Suggett et al., 2003). The lower α_*ETR*_ and higher $ETR_{PSII}^{max}$ observed for NWP *Trichodesmium* resulted in a higher saturating light intensity, *E*_*K*_ (846 ± 472 μmol quanta m^–2^ s^–1^), when compared to the NIO-SL (524 ± 136 μmol quanta m^–2^ s^–1^). This suggests an acclimation to increased available irradiance for *Trichodesmium* in the NWP region as mentioned above. Variations of α_*ETR*_ and $ETR_{PSII}^{max}$ were observed as well when data were considered at the local scale. Specifically, the largest α_*ETR*_ in the NWP appeared at station K13, whereas in the NIO-SL significantly higher α_*ETR*_ and $ETR_{PSII}^{max}$ were found at DT-01. Platt et al. (1992) reported a significant positive response of the initial slope (α) of the photosynthesis-light curve to increase in nitrate concentration during the spring bloom in the western North Atlantic. The photosynthesis rate in saturating light ($P_{max}^{B}$) was also significantly higher during the spring bloom when compared with the oligotrophic phase (Platt et al., 1992). In addition, positive correlations between α_*ETR*_ and $ETR_{PSII}^{max}$ with Fe concentration have been reported for cultured *Trichodesmium* (Boatman et al., 2018a). Therefore, the variations in α_*ETR*_ and $ETR_{PSII}^{max}$ of *Trichodesmium* observed in this study may be associated with Fe supply, and increased Fe availability (e.g., from rapid Fe-recycling) is likely to enhance electron transport especially under low light condition (i.e., higher α_*ETR*_).

It is important, however, to note that variation of *Trichodesmium* photobiology between the two study regions observed here could also be explained by factors other than differences in Fe availability. It is well known for example, that *Trichodesmium* exhibits distinct physiological ecology between different clades or strains (Chappell and Webb, 2010; Hutchins et al., 2013; Delmont, 2021). While we were able to confirm that the same *Trichodesmium* species (*T. thiebautii*—likely belonging to clade I) was dominant in both Western Pacific and Indian Ocean samples, further identification to strain level was not possible in the absence of further genomics analysis being conducted. Previous work by Hutchins et al. (2013), for example, has demonstrated up to a sixfold range in carbon affinity between *Trichodesmium* strains, indicative of strain-specific physiological strategies possibly reflecting adaptive responses to different biogeochemical regimes (Rouco et al., 2016). As such, we cannot rule out the possibility that the presence of different strains of *T. thiebautii* between the two study regions could have contributed to the observed differences in photobiology as assessed by FRRf.

### Fast Repetition Rate Fluorometry-Based Estimation of Primary Productivity for *Trichodesmium*

Photosynthetic electron transport generates the energy and reductant required for carbon fixation (Hughes et al., 2018a). As such, FRRf-derived electron turnover rates can theoretically be used to quantify Chl-a specific rates of primary productivity (*P^Chl^*). From our FRRf data, we retrieved *P^Chl^* values that ranged from 7.8 to 21.1 mgC mg Chl-a^–1^ h^–1^ with an average of 11.9 mgC mg Chl-a^–1^ h^–1^ (Table 5) and found that on average the NWP region was slightly more productive than the NIO-SL region. Notably, the station exhibiting the highest *P^Chl^* (K13) also had the highest PSII maximum photochemical efficiency (i.e., *F*_*v*_/*F*_*m*_) and the lowest values of non-photochemical quenching (*NPQ*_*NSV*_). In contrast, the lowest *P^Chl^* value recorded (station DM2-2) was accompanied by the lowest *F*_*v*_/*F*_*m*_ and highest *NPQ*_*NSV*_values. This observation further suggests that Fe is a main limiting factor for photosynthetic activity (and thus overall productivity) of *Trichodesmium* (Carpenter et al., 2004; Küpper et al., 2008; Rochelle-Newall et al., 2014).

Our reported range of *P^Chl^* is somewhat larger than previously reported rates of *in-situ* productivity in the Pacific Ocean, which ranged from 0.2 to 7.6, mgC mg Chl-a^–1^ h^–1^, with an average of 3.3 mgC mg Chl-a^–1^ h^–1^ (see Masotti et al., 2007). Whether this discrepancy reflects true differences in productivity between regions/studies or inherent uncertainties in scaling ETR to carbon-based photosynthetic rates is unclear. Deriving *P^Chl^* from FRRf data requires estimates of both (i) the number of PSII reaction centers per Chl-a (*n*_*PSII*_) and (ii) the proportion of photosynthetic electrons invested into C fixation (the so-called electron requirement for carbon fixation, Φ_*e*,*C*_) (Suggett et al., 2010; Oxborough et al., 2012; Lawrenz et al., 2013). Here, we used an estimated value for *n*_*PSII*_ of 0.027 as reported for *Trichodesmium* by Richier et al. (2012), a value which is an order of magnitude greater than typically assumed for cyanobacteria (0.003 RCII per Chl-a; Kolber and Falkowski, 1993). Use of an assumed *n*_*PSII*_ value is common in FRRf-based studies due to the complexity of directly measuring PSII content requiring use of oxygen flash yields which are often impractical to perform during field campaigns (Lawrenz et al., 2013). Laboratory studies have shown that *n*_*PSII*_ may exhibit physiological plasticity within and between phytoplankton taxa, and as such, our use of an assumed value likely introduces a degree of uncertainty in our *P^Chl^* values.

Since light intensities were relatively similar across sampling locations at the time of sampling, *P^Chl^* variability was largely determined by ETR rather than _*e,C*_ for our dataset (*r*^2^ = 0.95, *p* < 0.01). As such, at face value it seems unlikely our calculated Φ_*e*,*C*_ could contribute significantly to possible overestimation of *P^Chl^* values. Daily variation in the ETR-light curve of *Trichodesmium* can also affect the estimation of *P^Chl^* (Schuback et al., 2016)—although in this study, instantaneous PAR was lower than saturating light intensity (*E*_*K*_), and thus variation of *P^Chl^* was largely explained by α_*ETR*_ rather than $ETR_{PSII}^{max}$. However, it is also important to consider that the diazotrophic status of *Trichodesmium* spp. may complicate the estimation of Φ_*e*,*C*_ – a parameter that effectively sums the net distribution of photosynthetic electrons between C-fixing and non-C-fixing pathways (Hughes et al., 2018a). Fixing atmospheric N_2_ is energetically expensive, costing a minimum of 5 e^–^ plus and 16 mol ATP per mol N_2_-fixed (Boatman et al., 2018b). The additional e^–^ flow and ATP needed for assimilating N_2_ inevitably diverts electrons away from C fixation and consequently leads to increased Φ_*e*,*C*_ (Hughes et al., 2018a). In this study, we did, however, calculate Φ_*e*,*C*_ using an algorithm derived from *Trichodesmium* grown with N_2_ as the sole N source (see Boatman et al., 2018b) and thus have likely inherently accounted for such increased redirection of electrons/ATP away from C fixation. Critically, however, the temporal separation between N_2_ fixation and photosynthesis has previously been observed for *Trichodesmium* in the field (Berman-Frank et al., 2001), and thus it is likely that Φ_*e*,*C*_ exhibits variability over the photoperiod. Consequently, the values for *P^Chl^* reported here should be interpreted with care considering the magnitude of N fixation was not simultaneously quantified. Future efforts to derive *P^Chl^* for *Trichodesmium* using FRRf would benefit from parallel measurement of ETR and C fixation (e.g., Hughes et al., 2020) but also quantification of N_2_-fixation activity in order to better understand separate biological and methodological influence on observed *P^Chl^* variability.

## Concluding Remarks

*Trichodesmium* is a major contributor to marine N_2_ fixation, providing bioavailable N for primary production (Richier et al., 2012). While FRRf potentially provides a novel capability to non-invasively probe the photosynthetic activity of *Trichodesmium*, a major current constraint against widespread application is whether and how applicable this approach is for this and other phytoplankton taxa and/or under certain specific physiological conditions, such as iron limitation (Hughes et al., 2018a). Utilizing multi-wavelength FRRf, we successfully investigated natural *Trichodesmium* populations in NWP and North Indian Ocean, highlighting a possible role for Fe availability on *Trichodesmium* regional photobiological characteristics. Our findings further highlight that FRRf instruments equipped with blue LEDs only fail to adequately drive PSII reaction center closure in *Trichodesmium* samples, resulting in underestimation of *F*_*v*_/*F*_*m*_, σ_*PSII*_, and ETRs (e.g., Raateoja et al., 2004).

We further applied an algorithm to estimate primary productivity of *Trichodesmium* from FRRf-derived fluorescence parameters. Importantly, however, accurate retrieval of carbon fixation rates for *Trichodesmium* populations from FRRf data will likely depend on improved knowledge of how *n*_*PSII*_ and _*e,C*_ vary within and between major *Trichodesmium* clades, and how diel variability of photophysiology affect primary productivity over daily scales (e.g., Berman-Frank et al., 2001).

## Data Availability Statement

The original contributions presented in the study are included in the article/Supplementary Material, further inquiries can be directed to the corresponding author/s.

## Author Contributions

YZ collected the samples, analyzed the FRRf data, developed the method in this study, and wrote the first draft of this manuscript. YF and ZW helped to collect *Trichodesmium* samples in the NWP and Chl-a measurements. TB and DH were responsible for the partial FRRf calculation, data analyses, and manuscript revision. QH, RZ, and QM made the contribution on results interpretation and manuscript writing. MW contributed to the FRRf in field measurements and manuscript revision. YF and ZJ assisted with taxonomic identification of *Trichodesmium* samples. ZJ and LS were also responsible for interpreting nutrient and phytoplankton community results. PD and WP contributed to the FRRf *in situ* measurements and *Trichodesmium* sample collection in the NIO-SL. JZ and FC developed the research design and made the contribution on manuscript revision. All authors contributed to the article and approved the submitted version.

## Conflict of Interest

The authors declare that the research was conducted in the absence of any commercial or financial relationships that could be construed as a potential conflict of interest.

## Publisher’s Note

All claims expressed in this article are solely those of the authors and do not necessarily represent those of their affiliated organizations, or those of the publisher, the editors and the reviewers. Any product that may be evaluated in this article, or claim that may be made by its manufacturer, is not guaranteed or endorsed by the publisher.

## References

[B1] Alderkamp A. C. de Baar H. J. Visser R. J. Arrigo K. R. (2010). Can photoinhibition control phytoplankton abundance in deeply mixed water columns of the Southern Ocean? *Limnol. Oceanogr.* 55 1248–1264. 10.4319/lo.2010.55.3.1248

[B2] Behrenfeld M. J. Milligan A. J. (2013). Photophysiological expressions of iron stress in phytoplankton. *Annu. Rev. Mar. Sci.* 5 217–246. 10.1146/annurev-marine-121211-172356 22881354

[B3] Behrenfeld M. J. Bale A. J. Kolber Z. S. Aiken J. Falkowski P. G. (1996). Confirmation of iron limitation of phytoplankton photosynthesis in the equatorial Pacific Ocean. *Nature* 383 508–511. 10.1038/383508a0

[B4] Behrenfeld M. Worthington K. Sherrell R. Chavez F. P. Strutton P. McPhaden M. (2006). Controls on tropical Pacific Ocean productivity revealed through nutrient stress diagnostics. *Nature* 442 1025–1028. 10.1038/nature05083 16943835

[B5] Berman-Frank I. Lundgren P. Chen Y. B. Küpper H. Kolber Z. Bergman B. (2001). Segregation of nitrogen fixation and oxygenic photosynthesis in the marine cyanobacterium *Trichodesmium*. *Science* 294 1534–1537. 10.1126/science.1064082 11711677

[B6] Berman-Frank I. Quigg A. Finkel Z. V. Irwin A. J. Haramaty L. (2007). Nitrogen-fixation strategies and Fe requirements in cyanobacteria. *Limnol. Oceanogr.* 52 2260–2269. 10.4319/lo.2007.52.5.2260

[B7] Boatman T. G. Davey P. A. Lawson T. Geider R. J. (2018b). The physiological cost of diazotrophy for *Trichodesmium erythraeum* IMS101. *PLoS One* 13:e0195638. 10.1371/journal.pone.0195638 29641568 PMC5895029

[B8] Boatman T. G. Oxborough K. Gledhill M. Lawson T. Geider R. J. (2018a). An integrated response of *Trichodesmium erythraeum* IMS101 growth and photo-physiology to Iron, CO_2_, and light intensity. *Front. Microbiol.* 9:624. 10.3389/fmicb.2018.00624 29755417 PMC5932364

[B9] Bowman T. E. Lancaster L. J. (1965). A bloom of the planktonic blue-green alga, *Trichodesmium erythraeum*, in the Tonga Islands. *Limnol. Oceanogr.* 10 291–293. 10.4319/lo.1965.10.2.0291

[B10] Breitbarth E. Oschlies A. LaRoche J. (2007). Physiological constraints on the global distribution of *Trichodesmium*–effect of temperature on diazotrophy. *Biogeosciences* 4 53–61. 10.5194/bg-4-53-2007

[B11] Browning T. J. Bouman H. A. Moore C. M. Schlosser C. Tarran G. A. Woodward E. M. S. (2014a). Nutrient regimes control phytoplankton ecophysiology in the South Atlantic. *Biogeosciences* 11 463–479. 10.5194/bg-11-463-2014

[B12] Browning T. J. Bouman H. A. Moore C. M. (2014b). Satellite-detected fluorescence: decoupling nonphotochemical quenching from iron stress signals in the South Atlantic and Southern Ocean. *Global Biogeochem. Cycles* 28 510–524. 10.1002/2013GB004773

[B13] Browning T. J. Liu X. Zhang R. Wen Z. Liu J. Zhou Y. (2021). Nutrient co-limitation in the subtropical Northwest Pacific. *Limnol. Oceanogr Lett.* 7 5–61. 10.1002/lol2.10205

[B14] Cai X. Gao K. Fu F. Campbell D. A. Beardall J. Hutchins D. A. (2015). Electron transport kinetics in the diazotrophic cyanobacterium *Trichodesmium* spp. grown across a range of light levels. *Photosynth. Res.* 124 45–56. 10.1007/s11120-015-0081-5 25616859

[B15] Campbell D. Hurry V. Clarke A. K. Gustafsson P. Öquist G. (1998). Chlorophyll fluorescence analysis of cyanobacterial photosynthesis and acclimation. *Microbiol. Mol. Biol. Rev.* 62 667–683. 10.1128/MMBR.62.3.667-683.1998 9729605 PMC98930

[B16] Capone D. G. Burns J. A. Montoya J. P. Subramaniam A. Mahaffey C. Gunderson T. (2005). Nitrogen fixation by *Trichodesmium* spp.: an important source of new nitrogen to the tropical and subtropical North Atlantic Ocean. *Global Biogeochem. Cycles* 19:GB2024. 10.1029/2004GB002331

[B17] Capone D. G. Zehr J. P. Paerl H. W. Berman B. Carpenter E. J. (1997). *Trichodesmium*, a globally significant marine cyanobacterium. *Science* 276 1221–1229. 10.1126/science.276.5316.1221

[B18] Carpenter E. J. Subramaniam A. Capone D. G. (2004). Biomass and primary productivity of the cyanobacterium *Trichodesmium* spp. in the tropical N Atlantic ocean. *Deep Sea Res. I Oceanogr. Res. Papers* 51 173–203. 10.1016/j.dsr.2003.10.006

[B19] Carvalho F. Gorbunov M. Y. Oliver M. J. Haskins C. Aragon D. Kohut J. T. (2020). FIRe glider: mapping in situ chlorophyll variable fluorescence with autonomous underwater gliders. *Limnol. Oceanogr Lett.* 18 531–545. 10.1002/lom3.10380

[B20] Cermeño P. Marañón E. Pérez V. Serret P. Fernández E. Castro C. G. (2006). Phytoplankton size structure and primary production in a highly dynamic coastal ecosystem (Ría de Vigo, NW-Spain): seasonal and short-time scale variability. *Estuar. Coast. Shelf Sci.* 67 251–266. 10.1016/j.ecss.2005.11.027

[B21] Chappell P. D. Webb E. A. (2010). A molecular assessment of the iron stress response in the two phylogenetic clades of *Trichodesmium*. *Environ. Microbiol.* 12 13–27. 10.1111/j.1462-2920.2009.02026.x 19708870

[B22] Chappell P. D. Moffett J. W. Hynes A. M. Webb E. A. (2012). Molecular evidence of iron limitation and availability in the global diazotroph *Trichodesmium*. *ISME J.* 6 1728–1739. 10.1038/ismej.2012.13 22402399 PMC3498915

[B23] Cheah W. McMinn A. Griffiths F. B. Westwood K. J. Wright S. W. Molina E. (2011). Assessing Sub-Antarctic Zone primary productivity from fast repetition rate fluorometry. *Deep Sea Res. II Top. Stud. Oceanogr.* 58 2179–2188. 10.1016/j.dsr2.2011.05.023

[B24] Ciotti A. Lewis M. Cullen J. (2002). Assessment of the relationship between dominant cell size in natural phytoplankton communities and the spectral shape of the absorption coefficient. *Limnol. Oceanogr.* 47 404–417. 10.4319/lo.2002.47.2.0404

[B25] Cleveland J. S. Weidemann A. D. (1993). Quantifying absorption by aquatic particles: a multiple scattering correction for glass-fiber filters. *Limnol. Oceanogr.* 38 1321–1327.

[B26] Croteau D. Guérin S. Bruyant F. Ferland J. Campbell D. A. Babin M. (2021). Contrasting nonphotochemical quenching patterns under high light and darkness aligns with light niche occupancy in Arctic diatoms. *Limnol. Oceanogr.* 66 S231–S245. 10.1002/lno.11587

[B27] Cullen J. J. Davis R. F. (2003). The blank can make a big difference in oceanographic measurements. *Limnol. Oceanogr. Bull.* 12 29–35. 10.1002/lob.200312229

[B28] Delmont T. O. (2021). Discovery of nondiazotrophic *Trichodesmium* species abundant and widespread in the open ocean. *Proc. Natl. Acad. Sci. U. S. A.* 118:e2112355118. 10.1073/pnas.2112355118 34750267 PMC8609553

[B29] Dore J. E. Letelier R. M. Church M. J. Lukas R. Karl D. M. (2008). Summer phytoplankton blooms in the oligotrophic North Pacific Subtropical Gyre: historical perspective and recent observations. *Prog. Oceanogr.* 76 2–38. 10.1016/j.pocean.2007.10.002

[B30] Falkowski P. G. Lin H. Gorbunov M. Y. (2017). What limits photosynthetic energy conversion efficiency in nature? Lessons from the oceans. *Philos. Trans. R. Soc. Lond. B Biol. Sci.* 372:20160376. 10.1098/rstb.2016.0376 28808095 PMC5566876

[B31] Falkowski P. G. Ziemann D. Kolber Z. Bienfang P. K. (1991). Role of eddy pumping in enhancing primary production in the ocean. *Nature* 352 55–58. 10.1038/352055a0

[B32] Frischkorn K. R. Krupke A. Guieu C. Louis J. Rouco M. Salazar Estrada A. E. (2018). *Trichodesmium* physiological ecology and phosphate reduction in the western tropical South Pacific. *Biogeosciences* 15 5761–5778. 10.5194/bg-15-5761-2018

[B33] Fujiki T. Hosaka T. Kimoto H. Ishimaru T. Saino T. (2008). *In situ* observation of phytoplankton productivity by an underwater profiling buoy system: use of fast repetition rate fluorometry. *Mar. Ecol. Prog. Ser.* 353 81–88. 10.3354/meps07151

[B34] Fujita Y. Shimura S. (1974). Phycoerythrin of the marine bluegreen alga *Trichodesmium thiebautii*. *Plant Cell Physiol.* 15 939–942.

[B35] Garside C. (1982). A chemiluminescent technique for the determination of nanomolar concentrations of nitrate and nitrite in seawater. *Mar. Chem.* 11 159–167. 10.1016/0304-4203(82)90039-1

[B36] Geider R. J. Greene R. M. Kolber Z. Macintyre H. L. Falkowski P. G. (1993). Fluorescence assessment of the maximum quantum efficiency of photosynthesis in the western North Atlantic. *Deep Sea Res I Oceanogr. Res. Papers* 40 1205–1224. 10.1016/0967-0637(93)90134-O

[B37] Gorbunov M. Y. Kolber Z. S. Falkowski P. G. (1999). Measuring photosynthetic parameters in individual algal cells by Fast Repetition Rate fluorometry. *Photosynth. Res.* 62 141–153. 10.1023/A:1006360005033

[B38] Gorbunov M. Y. Kolber Z. S. Lesser M. P. Falkowski P. G. (2001). Photosynthesis and photoprotection in symbiotic corals. *Limnol. Oceanogr.* 46 75–85. 10.4319/lo.2001.46.1.0075

[B39] Gorbunov M. Y. Kuzminov F. I. Fadeev V. V. Kim J. D. Falkowski P. G. (2011). A kinetic model of non-photochemical quenching in cyanobacteria. *Biochim. Biophys. Acta. Bioenerg.* 1807 1591–1599. 10.1016/j.bbabio.2011.08.009 21907180

[B40] Gorbunov M. Y. Shirsin E. Nikonova E. Fadeev V. V. Falkowski P. G. (2020). A multi-spectral fluorescence induction and relaxation (FIRe) technique for physiological and taxonomic analysis of phytoplankton communities. *Mar. Ecol. Prog. Ser.* 644 1–13. 10.3354/meps13358

[B41] Hopkinson B. M. Mitchell B. G. Reynolds R. A. Wang H. Selph K. E. Measures C. I. (2007). Iron limitation across chlorophyll gradients in the southern Drake Passage: phytoplankton responses to iron addition and photosynthetic indicators of iron stress. *Limnol. Oceanogr.* 52 2540–2554.

[B42] Hoppe C. J. Holtz L. M. Trimborn S. Rost B. (2015). Ocean acidification decreases the light use efficiency in an Antarctic diatom under dynamic but not constant light. *New Phytol.* 207 159–171. 10.1111/nph.13334 25708812 PMC4950296

[B43] Horton P. A. Ruban V. Walters R. G. (1996). Regulation of light harvesting in green plants. *Annu. Rev. Plant Physiol. Plant Mol. Biol.* 47 655–684. 10.1146/annurev.arplant.47.1.655 15012304

[B44] Houliez E. Simis S. Nenonen S. Ylöstalo P. Seppälä J. (2017). Basin-scale spatio-temporal variability and control of phytoplankton photosynthesis in the Baltic Sea: the first multiwavelength fast repetition rate fluorescence study operated on a ship-of-opportunity. *J. Mar. Syst.* 169 40–51. 10.1016/j.jmarsys.2017.01.007

[B45] Huang R. X. Russell S. (1994). Ventilation of the subtropical North Pacific. *J. Phys. Oceanogr.* 24 2589–2605. 10.1126/sciadv.abd1654 33298448 PMC7725469

[B46] Hughes D. J. Campbell D. A. Doblin M. A. Kromkamp J. C. Lawrenz E. Moore C. M. (2018a). Roadmaps and detours: active chlorophyll- a assessments of primary productivity across marine and freshwater systems. *Environ. Sci. Technol.* 52 12039–12054. 10.1021/acs.est.8b03488 30247887

[B47] Hughes D. J. Varkey D. Doblin M. A. Ingleton T. Mcinnes A. Ralph P. J. (2018b). Impact of nitrogen availability upon the electron requirement for carbon fixation in Australian coastal phytoplankton communities. *Limnol. Oceanogr.* 63 1891–1910. 10.1002/lno.10814

[B48] Hughes D. J. Crosswell J. R. Doblin M. A. Oxborough K. Ralph P. J. Varkey D. (2020). Dynamic variability of the phytoplankton electron requirement for carbon fixation in eastern Australian waters. *J. Mar. Res.* 202:103252. 10.1016/j.jmarsys.2019.103252

[B49] Hughes D. J. Giannini F. C. Ciotti A. M. Doblin M. A. Ralph P. J. Varkey D. (2021). Taxonomic variability in the electron requirement for carbon fixation across marine phytoplankton. *J. Phycol.* 57 111–127. 10.1111/jpy.13068 32885422

[B50] Hutchins D. A. Fu F. X. Webb E. A. Walworth N. Tagliabue A. (2013). Taxon-specific response of marine nitrogen fixers to elevated carbon dioxide concentrations. *Nat. Geosci.* 6 790–795. 10.1038/ngeo1858

[B51] Hynes A. M. Chappell P. D. Dyhrman S. T. Doney S. C. Webb E. A. (2009). Cross-basin comparison of phosphorus stress and nitrogen fixation in *Trichodesmium*. *Limnol. Oceanogr.* 54 1438–1448. 10.4319/lo.2009.54.5.1438

[B52] Ihnken S. Kromkamp J. C. Beardall J. (2011). Photoacclimation in *Dunaliella tertiolecta* reveals a unique NPQ pattern upon exposure to irradiance. *Photosynth. Res.* 110 123–137. 10.1007/s11120-011-9709-2 22101577 PMC3224225

[B53] Jiang Z. Li H. Zhai H. Zhou F. Chen Q. Chen J. (2018). Seasonal and spatial changes in *Trichodesmium* associated with physicochemical properties in East China Sea and southern Yellow Sea. *J. Geophys. Res. Biogeosci.* 123 509–530. 10.1002/2017JG004275

[B54] Karl D. M. Letelier R. Tupas L. Dore J. Christian J. Hebel D. (1997). The role of nitrogen fixation in biogeochemical cycling in the subtropical North Pacific Ocean. *Nature* 388 533–538. 10.1038/41474

[B55] Kazama T. Hayakawa K. Kuwahara V. S. Shimotori K. Imai A. Komatsu K. (2021). Development of photosynthetic carbon fixation model using multi-excitation wavelength fast repetition rate fluorometry in Lake Biwa. *PLoS One* 16:e0238013. 10.1371/journal.pone.0238013 33529253 PMC7853527

[B56] Kolber Z. S. Prášil O. Falkowski P. G. (1998). Measurement of variable chlorophyll fluorescence using fast repetition rate techniques: defining methodology and experimental protocols. *Biochim. Biophys. Acta* 1367 88–106. 10.1016/s0005-2728(98)00135-2 9784616

[B57] Kolber Z. Falkowski P. G. (1993). Use of active fluorescence to estimate phytoplankton photosynthesis in situ. *Limnol. Oceanogr.* 38 1646–1665. 10.4319/lo.1993.38.8.1646

[B58] Kolber Z. Wyman K. D. Falkowski P. G. (1990). Natural variability in photosynthetic energy conversion efficiency; a field study in the Gulf of Maine. *Limnol. Oceanogr.* 35 72–79. 10.4319/lo.1990.35.1.0072

[B59] Kolber Z. Zehr J. Falkowski P. G. (1988). Effects of growth irradiance and nitrogen limitation on photosynthetic energy conversion in photosystem II. *Plant Physiol.* 88 923–929. 10.1104/pp.88.3.923 16666405 PMC1055683

[B60] Krause G. H. Weis E. (1991). Chlorophyll fluorescence and photosynthesis: the basics. *Annu. Rev. Plant Physiol. Plant Mol. Biol.* 42 313–349. 10.1146/annurev.pp.42.060191.001525

[B61] Kromkamp J. C. Forster R. M. (2003). The use of variable fluorescence measurements in aquatic ecosystems: differences between multiple and single turnover measuring protocols and suggested terminology. *Eur. J. Phycol.* 38 103–112. 10.1080/0967026031000094094

[B62] Kulk G. van de Poll W. H. Visser R. J. W. Buma A. G. J. (2013). Low nutrient availability reduces high-irradiance-induced viability loss in oceanic phytoplankton. *Limnol. Oceangr.* 58 1747–1760. 10.4319/lo.2013.58.5.1747

[B63] Küpper H. Šetlík I. Seibert S. Prášil O. Šetlikova E. Strittmatter M. (2008). Iron limitation in the marine cyanobacterium *Trichodesmium* reveals new insights into regulation of photosynthesis and nitrogen fixation. *New Phytol.* 179 784–798. 10.1111/j.1469-8137.2008.02497.x 18513224

[B64] Lavaud J. Six C. Campbell D. A. (2016). Photosystem II repair in marine diatoms with contrasting photophysiologies. *Photosynth. Res.* 127 189–199. 10.1007/s11120-015-0172-3 26156125

[B65] Lawrenz E. Silsbe G. Capuzzo E. Ylöstalo P. Forster R. M. Simis S. G. (2013). Predicting the electron requirement for carbon fixation in seas and oceans. *PLoS One* 8:e58137. 10.1371/journal.pone.0058137 23516441 PMC3596381

[B66] Liu Z. Lian Q. Zhang F. Wang L. Li M. Bai X. (2017). Weak thermocline mixing in the North Pacific low-latitude western boundary current system. *Geophys. Res. Lett.* 44 10,530–10,539.

[B67] Ma J. Yuan D. Liang Y. (2008). Sequential injection analysis of nanomolar soluble reactive phosphorus in seawater with HLB solid phase extraction. *Mar. Chem.* 111 151–159. 10.1016/j.marchem.2008.04.011

[B68] Masotti I. Ruiz-Pino D. Le Bouteiller A. (2007). Photosynthetic characteristics of *Trichodesmium* in the southwest Pacific Ocean: importance and significance. *Mar. Ecol. Prog. Ser.* 338 47–59. 10.3354/meps338047

[B69] McKew B. A. Davey P. Finch S. J. Hopkins J. Lefebvre S. C. Metodiev M. V. (2013). The trade-off between the light-harvesting and photoprotective functions of fucoxanthin-chlorophyll proteins dominates light acclimation in Emiliania huxleyi (clone CCMP 1516). *New Phytol.* 200 74–85. 10.1111/nph.12373 23790241

[B70] Mills M. Ridame C. Davey M. Roche J. Geider J. R. (2004). Iron and phosphorus co-limit nitrogen fixation in the eastern tropical North Atlantic. *Nature* 429 292–294. 10.1038/nature02550 15152251

[B71] Moore C. M. Lucas M. I. Sanders R. Davidson R. (2005). Basin-scale variability of phytoplankton bio-optical characteristics in relation to bloom state and community structure in the Northeast Atlantic. *Deep Sea Res. I Oceanogr. Res. Papers* 52 401–419. 10.1016/j.dsr.2004.09.003

[B72] Moore C. M. Mills M. M. Langlois R. Milne A. Achterberg E. P. Roche J. L. (2008). Relative influence of nitrogen and phosphorous availability on phytoplankton physiology and productivity in the oligotrophic sub-tropical North Atlantic Ocean. *Limnol. Oceanogr.* 53 291–305. 10.4319/lo.2008.53.1.0291

[B73] Moore C. M. Suggett D. J. Hickman A. E. Kim Y.-N. Tweddle J. F. Sharples J. (2006). Phytoplankton photoacclimation and photoadaptation in response to environmental gradients in a shelf sea. *Limnol. Oceanogr.* 51 936–949.

[B74] Mullineaux C. Tobin M. Jones G. (1997). Mobility of photosynthetic complexes in thylakoid membranes. *Nature* 390 421–424. 10.1016/j.molp.2017.09.019 29017828 PMC5683893

[B75] Olaizola M. LaRoche J. Kolber Z. Falkowski P. G. (1994). Nonphotochemical quenching and the diadinoxanthin cycle in a marine diatom. *Photosynth. Res.* 41 357–370. 10.1007/BF00019413 24310118

[B76] Oxborough K. (2013). *FastPro8 GUI and FRRf3 Systems Documentation.* West Molesey: Chelsea Technologies Group Ltd.

[B77] Oxborough K. Baker N. R. (1997). Resolving chlorophyll a fluorescence images of photosynthetic efficiency into photochemical and non-photochemical components–calculation of qP and Fv/Fm; without measuring Fo. *Photosynth. Res.* 54 135–142. 10.1023/A:1005936823310

[B78] Oxborough K. Moore C. M. Suggett D. J. Lawson T. Chan H. G. Geider R. J. (2012). Direct estimation of functional PSII reaction center concentration and PSII electron flux on a volume basis: a new approach to the analysis of Fast Repetition Rate fluorometry (FRRf) data. *Limnol. Oceanogr. Methods* 10 142–154. 10.4319/lom.2012.10.142

[B79] Papageorgiou G. Tsimilli-Michael M. Stamatakis K. (2007). The fast and slow kinetics of chlorophyll a fluorescence induction in plants, algae and cyanobacteria: a viewpoint. *Photosynth. Res.* 94 275–229. 10.1007/s11120-007-9193-x 17665151

[B80] Platt T. Gallegos C. L. Harrison W. G. (1980). Photoinhibition of photosynthesis in natural assemblages of marine phytoplankton. *J. Mar. Res.* 38 687–701.

[B81] Platt T. Sathyendranath S. Ulloa O. Harrison W. G. Hoepffner N. Goes J. (1992). Nutrient control of phytoplankton photosynthesis in the Western North Atlantic. *Nature* 356 229–231.

[B82] R Core Team (2019). *R: A Language and Environment for Statistical Computing.* Vienna: R Foundation for Statistical Computing.

[B83] Raateoja M. Seppala J. Ylostalo P. (2004). Fast repetition rate fluorometry is not applicable to studies of filamentous cyanobacteria from the Baltic Sea. *Limnol. Oceanogr.* 49 1006–1012.

[B84] Richier S. Macey A. I. Pratt N. J. Honey D. J. Moore C. M. Bibby T. S. (2012). Abundances of Iron-binding photosynthetic and nitrogen-fixing proteins of *Trichodesmium* both in culture and *In Situ* from the North Atlantic. *PLoS One* 7:e35571. 10.1371/journal.pone.0035571 22563465 PMC3341377

[B85] Robinson C. Suggett D. J. Cherukuru N. Ralph P. J. Doblin M. A. (2014). Performance of fast repetition rate fluorometry based estimates of primary productivity in coastal waters. *J. Mar. Syst.* 139 299–310. 10.1016/j.jmarsys.2014.07.016

[B86] Rochelle-Newall E. J. Ridame C. Dimier-Hugueney C. Helguen S. L. (2014). Impact of iron limitation on primary production (dissolved and particulate) and secondary production in cultured *Trichodesmium* sp. *Aquat. Microb. Ecol.* 72 143–153.

[B87] Rouco M. Haley S. T. Alexander H. Wilson S. T. Karl D. M. Dyhrman S. T. (2016). Variable depth distribution of *Trichodesmium* clades in the North Pacific Ocean. *Environ. Microbiol. Rep.* 8 1058–1066. 10.1111/1758-2229.12488 27753237

[B88] Roy S. Sathyendranath S. Bouman H. Platt T. (2013). The global distribution of phytoplankton size spectrum and size classes from their light-absorption spectra derived from satellite data. *Remote Sens. Environ.* 139 185–197. 10.1016/j.rse.2013.08.004

[B89] Ryan-Keogh T. J. Thomalla S. J. (2020). Deriving a proxy for iron limitation from chlorophyll fluorescence on buoyancy gliders. *Front. Mar. Sci.* 7:275. 10.3389/fmars.2020.00275

[B90] Schlitzer R. (2018). *Ocean Data View*. Available online at: http://odv.awi.de.

[B91] Schreiber U. Schliwa U. Bilger W. (1986). Continuous recording of photochemical and non-photochemical chlorophyll fluorescence quenching with a new type of modulation fluorometer. *Photosynth. Res.* 10 51–62. 10.1007/BF00024185 24435276

[B92] Schuback N. Flecken M. Maldonado M. T. Tortell P. D. (2016). Diurnal variation in the coupling of photosynthetic electron transport and carbon fixation in iron-limited phytoplankton in the NE subarctic Pacific. *Biogeosciences* 13 16803–16845. 10.5194/bgd-12-16803-2015

[B93] Schuback N. Schallenberg C. Duckham C. Maldonado M. T. Tortell P. D. (2015). Interacting effects of light and iron availability on the coupling of photosynthetic electron transport and CO_2_-assimilation in marine phytoplankton. *PLoS One* 10:e0133235. 10.1371/journal.pone.0133235 26171963 PMC4501554

[B94] Schuback N. Tortell P. D. Berman-Frank I. Campbell D. A. Ciotti A. Courtecuisse E. (2021). Single-turnover variable chlorophyll fluorescence as a tool for assessing phytoplankton photosynthesis and primary productivity: opportunities, caveats and recommendations. *Front. Mar. Sci.* 8:690607. 10.3389/fmars.2021.690607

[B95] Silsbe G. M. Oxborough K. Suggett D. J. Forster R. M. Ihnken S. Komárek O. (2015). Toward autonomous measurements of photosynthetic electron transport rates: an evaluation of active fluorescence-based measurements of photochemistry. *Limnol. Oceanogr. Methods* 13 138–155. 10.1002/lom3.10014

[B96] Simis S. G. H. Huot Y. Babin M. Seppälä J. Metsamaa L. (2012). Optimization of variable fluorescence measurements of phytoplankton communities with cyanobacteria. *Photosynth. Res.* 112 13–30. 10.1007/s11120-012-9729-6 22403036 PMC3324691

[B97] Smyth T. J. Pemberton K. L. Aiken J. Geider R. J. (2004). A methodology to determine primary production and phytoplankton photosynthetic parameters from Fast Repetition Rate Fluorometry. *J. Plankton Res.* 26 1337–1350.

[B98] Subramaniam A. Carpenter E. J. Karentz D. Falkowski P. G. (1999). Bio-optical properties of the marine diazotrophic cyanobacteria *Trichodesmium* spp.: I Absorption and photosynthetic action spectra. *Limnol. Oceanogr.* 44 608–617. 10.4319/lo.1999.44.3.0608

[B99] Suggett D. J. Kraay G. Holligan P. Davey M. Aiken J. Geider R. (2001). Assessment of photosynthesis in a spring cyanobacterial bloom by use of a fast repetition rate fluorometer. *Limnol. Oceanogr.* 46 802–810.

[B100] Suggett D. J. MacIntyre H. L. Geider R. J. (2004). Evaluation of biophysical and optical determinations of light absorption by photosystem II in phytoplankton. *Limnol. Oceanogr. Methods* 2 316–332.

[B101] Suggett D. J. MacIntyre H. L. Kana T. M. Geider R. J. (2009a). Comparing electron transport with gas exchange: parameterising exchange rates between alternative photosynthetic currencies for eukaryotic phytoplankton. *Aquat. Microb. Ecol.* 56 147–162.

[B102] Suggett D. J. Moore C. M. Hickman A. E. Geider R. J. (2009b). Interpretation of fast repetition rate (FRR) fluorescence: signatures of phytoplankton community structure versus physiological state. *Mar. Ecol. Prog. Ser.* 376 1–19.

[B103] Suggett D. J. Moore C. M. Geider R. J. (2010). “Estimating aquatic productivity from active fluorescence measurements,” in *Chlorophyll a Fluorescence in Aquatic Sciences: Methods and Applications*, eds Suggett D. Prášil O. Borowitzka M. (Berlin: Springer), 103–127.

[B104] Suggett D. J. Moore C. M. Marañón E. Omachi C. Varela R. A. Aiken J. (2006). Photosynthetic electron turnover in the tropical and subtropical Atlantic Ocean. *Deep Sea Res. II Top. Stud. Oceanogr.* 53 1573–1592.

[B105] Suggett D. J. Oxborough K. Baker N. R. MacIntyre H. L. Kana T. M. Geider R. J. (2003). Fast Repetition Rate and Pulse Amplitude Modulation chlorophyll a fluorescence measurements for assessment of photosynthetic electron transport in marine phytoplankton. *Eur. J. Phycol.* 38 371–384.

[B106] Timmermans K. R. Davey M. S. van der Wagt B. Snoek J. Geider R. J. Veldhuis M. J. (2001). Co-limitation by iron and light of Chaetoceros brevis, C. dichaeta and C. calcitrans (*Bacillariophyceae*). *Mar. Ecol. Prog. Ser.* 217 287–297.

[B107] Twining S. B. Rauschenberg S. Baer E. S. Lomas W. M. Martiny C. A. Antipova O. (2019). A nutrient limitation mosaic in the eastern tropical Indian Ocean. *Deep Sea Res. II Top. Stud. Oceanogr.* 166 125–140.

[B108] Udarbe-Walker M. J. B. Villanoy C. L. (2001). Structure of potential upwelling areas in the Philippines. *Deep Sea Res. I Oceanogr. Res. Papers* 48 1499–1518.

[B109] Vos A. D. Pattiaratchi C. B. Wijeratne E. M. S. (2014). Surface circulation and upwelling patterns around Sri Lanka. *Biogeosciences* 11 5909–5930.

[B110] Walworth N. G. Fu F.-X. Webb E. A. Saito M. A. Moran D. Mcllvin M. R. (2016). Mechanisms of increased *Trichodesmium* fitness under iron and phosphorus co-limitation in the present and future ocean. *Nat. Commun.* 7:12081. 10.1038/ncomms12081 27346420 PMC4931248

[B111] Wang S. Ishizaka J. Yamaguchi H. Tripathy S. C. Hayashi M. Xu Y. J. (2014). Influence of the Changjiang River on the light absorption properties of phytoplankton from the East China Sea. *Biogeosciences* 11 1759–1773.

[B112] Wei Y. Chen Z. Guo C. Zhong Q. Wu C. Sun J. (2020). Physiological and ecological responses of photosynthetic processes to oceanic properties and phytoplankton communities in the oligotrophic Western Pacific Ocean. *Front. Microbiol.* 11:1774. 10.3389/fmicb.2020.01774 32849398 PMC7417450

[B113] Wei Y. Zhao X. Sun J. Liu H. (2019). Fast Repetition Rate Fluorometry (FRRF) derived phytoplankton primary productivity in the Bay of Bengal. *Front. Microbiol.* 10:1164. 10.3389/fmicb.2019.01164 31244786 PMC6544007

[B114] Welschmeyer N. A. (1994). Fluorometric analysis of chlorophyll-a in the presence of chlorophyll-b and phaeopigments. *Limnol. Oceanogr.* 39 1985–1992.

[B115] Wu J. F. Sunda W. Boyle E. A. Karl D. M. (2000). Phosphate depletion in the western North Atlantic Ocean. *Science* 289 759–762. 10.1126/science.289.5480.759 10926534

[B116] Zhu Y. Ishizaka J. Tripathy S. C. Wang S. Mino Y. Matsuno T. (2016). Variation of the photosynthetic electron transfer rate and electron requirement for daily net carbon fixation in Ariake Bay. *Japan J. Oceanogr.* 72 761–776. 10.1007/s10872-016-0370-4

[B117] Zhu Y. Ishizaka J. Tripathy S. C. Wang S. Sukigara C. Goes J. (2017). Relationship between light, community composition and the electron requirement for carbon fixation in natural phytoplankton. *Mar. Ecol. Prog. Ser.* 580 83–100. 10.3354/meps1231

[B118] Zhu Y. Suggett D. J. Liu C. He J. Lin L. Le F. (2019). Primary productivity dynamics in the summer Arctic Ocean confirms broad regulation of the electron requirement for carbon fixation by light-phytoplankton community interaction. *Front. Mar. Sci.* 6:275. 10.3389/fmars.2019.00275

