# Supplementary material for: Exploring Variability of Trichodesmium Photophysiology Using Multi-Excitation Wavelength Fast Repetition Rate Fluorometry
Source: Front Microbiol. 2022 Apr 8;13:813573. doi: 10.3389/fmicb.2022.813573 (PMC9026164; doi:10.3389/fmicb.2022.813573)
Supplement: Supplementary file 1 [file Data_Sheet_1.docx]

Supplementary Material

Exploring photophysiological variability in natural populations of *Trichodesmium* using multiexcitation wavelength Fast Repetition Rate fluorometry

**Yuanli Zhu*, Yuanyuan Feng, Thomas J. Browning, Zuozhu Wen, David J. Hughes, Qiang Hao, Ruifeng Zhang, Qicheng Meng, Mark L. Wells, Zhibing Jiang, P.A.K.N Dissanayake, W.N.C. Priyadarshani, Lu Shou, Jiangning Zeng, Fei Chai**

*** Correspondence: Yuanli Zhu**: [ylzhu@sio.org.cn](mailto:ylzhu@sio.org.cn)

# Supplementary Figures and Tables


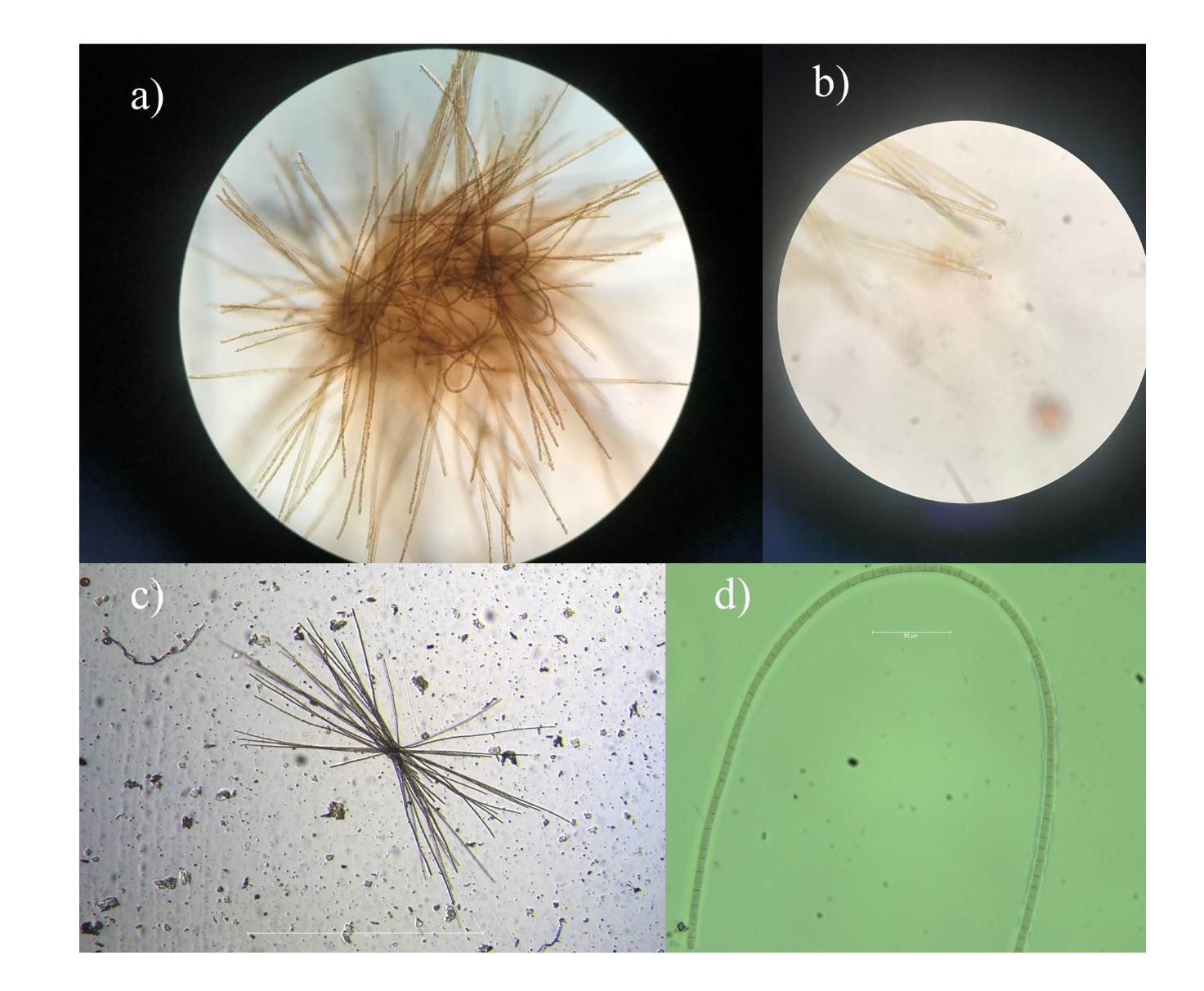


**Supplementary Figure 1.** Microscopic photos of *Trichodesmium* (T*. thiebautii* ) in the Northwest Pacific (A, B) and North Indian Ocean in the vicinity of Sri Lanka (C, D)


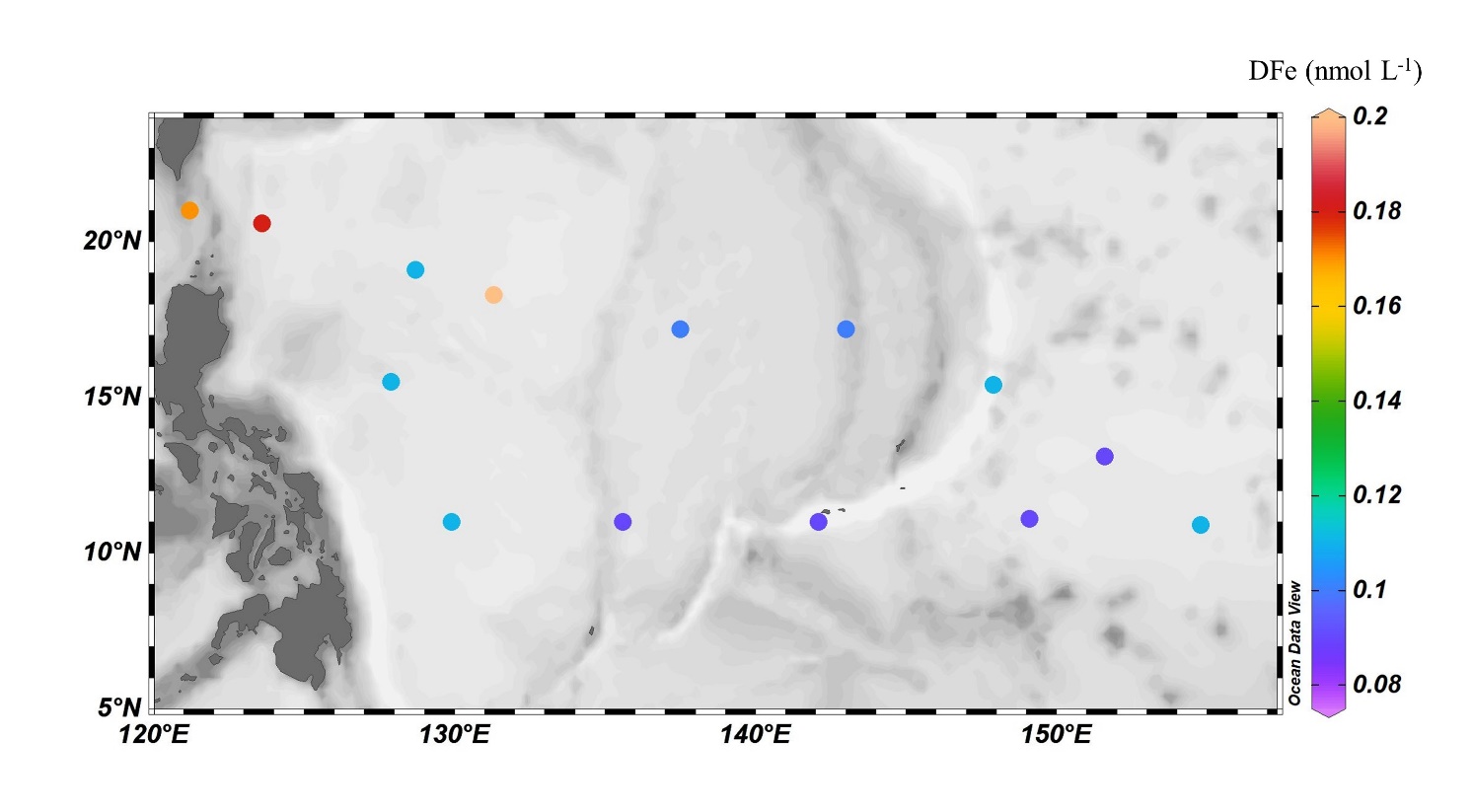
 **Supplementary Figure 2.** Dissolved iron concentration (DFe) at surface water during GP09 cruise, and the data is from Browning et al (2021).

**Supplementary Table 1.** *Prochlorococcus* proportion of the picophytoplankton group (<3 µm) at sampling sites of Northwest Pacific (NWP) and Sri Lanka coast (SLC) waters measured by FlowCytomer

| Region | Stn. | *Prochlorococcus* % |
| --- | --- | --- |
|  | K4 | 95.6 |
|  | K5 | 95.5 |
|  | K7 | 95.2 |
|  | K8 | 96.4 |
| NWP | K10 | 97.1 |
|  | K12 | 98.2 |
|  | K13 | 98.5 |
|  | K14 | 97.9 |
|  | K14a | 94.5 |
|  | **Mean** | **96.8**  **(1.4)** |
|  | DT-01 | 72.2 |
| SLC | DM2-2 | 89.3 |
|  | SL1-2 | 90.2 |
|  | **Mean** | **83.9**  **(8.2)** |

## REFERENCE

Browning, T. J., Liu, X., Zhang, R., Wen, Z., Liu, J., Zhou, Y., et al. (2021). Nutrient co‐limitation in the subtropical Northwest Pacific. *Limnol. Oceanogr Lett.* https://doi.org/10.1002/lol2.10205
